# Genetic dissection of apricot fruit skin color (*Prunus armeniaca* L.) using SNP and SSR molecular markers

**DOI:** 10.1007/s11032-026-01674-5

**Published:** 2026-05-22

**Authors:** Germán Ortuño-Hernández, Lorenzo Bergonzoni, Stefano Tartarini, Mónica Moya-Andreo, Jesús López-Alcolea, David Ruiz, Pedro Martínez-Gómez, Luca Dondini, Juan Alfonso Salazar

**Affiliations:** 1 Fruit Breeding Group, Department of Plant Breeding, CEBAS-CSIC (Centro de Edafología y Biología Aplicada del Segura-Consejo Superior de Investigaciones Científicas), Campus Universitario Espinardo, 30100 Murcia, Spain; 2 Department of Agricultural and Food Sciences (DISTAL), University of Bologna, Bologna, Italy

**Keywords:** Apricot, Carotenoids, Color, HRM, MAS, SSR, SNP

## Abstract

Fruit skin color is a key quality trait in apricot, strongly influencing consumer preference and market value. However, phenotypic evaluation of this trait is time-consuming and highly dependent on environmental conditions and harvest timing. This study aimed to develop reliable molecular markers associated with apricot skin color to support marker-assisted selection in breeding programs. A major skin color QTL located on chromosome 3 was explored, leading to the identification of an AP2/ERF domain–containing gene involved in carotenoid regulation. Within this gene, both SNP and SSR polymorphisms were characterized. High-resolution melting (HRM) analysis validated a highly significant SNP associated with fruit skin color in a segregating ‘Goldrich’ × ‘Currot’ population and in a panel of 57 apricot cultivars. The HRM-based SNP marker accurately discriminated between orange- and yellow-skinned fruits, reaching 100% efficiency in the segregating population and 89.5% in the cultivar set. In parallel, SSR markers developed within the AP2/ERF gene revealed allelic variation associated with skin color, with one SSR marker achieving an efficiency of 93.9%. Allelic and protein sequence reconstruction demonstrated that variation in polyalanine tract length and non-synonymous amino acid substitutions generate functional diversity in the AP2/ERF protein. Notably, this analysis indicated that a marker based on SNP4 (S3_22924316), which directly affects the protein sequence, may provide enhanced discriminatory power. comparable to that previously observed for the SSR-based marker. Overall, these results provide both mechanistic insight into apricot color regulation and practical molecular tools for efficient breeding.

**Supplementary Information:**

The online version contains supplementary material available at 10.1007/s11032-026-01674-5.

## Introduction

Apricot (*Prunus armeniaca* L.) is a diploid (2n = 16) perennial fruit tree belonging to the *Rosaceae* family and the genus *Prunus*, which also includes economically important species such as peach, almond, plum, and cherry (Salazar et al. 2026b). Apricot fruit is highly valued for its nutritional and sensory attributes, including high levels of carotenoids, phenolic compounds, vitamins, and sugars, which contribute to its characteristic flavor, color, and antioxidant capacity (Fratianni et al. 2018). Due to these qualities, apricot is consumed both as fresh fruit and in processed forms, particularly as dried fruit, juices, and preserves (Al-Soufi et al. 2022). Originating in Central Asia, apricot cultivation has expanded widely across temperate regions of the world, adapting to diverse climatic conditions and production systems (Hormaza et al. 2007). Globally, apricot production is concentrated in Mediterranean and Near Eastern regions. Turkey stands out as the leading producer worldwide, accounting for approximately 20.1% of global production in 2023, with a strong emphasis on dried apricot production (FAOSTAT, Production). In contrast, Spain has consolidated its position as one of the main exporters of fresh apricots, ranking first in export volume, with 77,589 tonnes exported in 2023 (FAOSTAT, Export).

Apricot breeding programs have traditionally focused on improving agronomic performance and fruit quality traits while addressing major phytosanitary challenges (Bassi and Audergon 2001; Llácer 2007; Ledbetter 2010). Among the primary objectives are resistance to Sharka disease, caused by Plum pox virus (PPV), and the incorporation of self-compatibility to ensure stable yields under variable pollination conditions (Burgos et al. 1997; Zhebentyayeva et al. 2011). To accelerate the selection process, molecular markers have been increasingly integrated into apricot breeding schemes (Vilanova et al. 2003). To date, the most widely implemented molecular markers in apricot are those linked to PPV resistance (Polo-Oltra et al. 2020) and self-compatibility (Vilanova et al. 2005), which are routinely used for marker-assisted selection at early developmental stages.

In recent years, advances in high-throughput sequencing technologies have dramatically expanded the genomic resources available for *Prunus* species (Aranzana et al. 2019). High-quality reference genomes have been published for peach (*Prunus persica*) (Verde et al. 2017), apricot (*Prunus armeniaca*) (Campoy et al. 2020), almond (*Prunus dulcis*) (D’Amico-Willman et al. 2022; Zhang et al. 2025), and sweet cherry (*Prunus avium*) (Shirasawa et al. 2017; Yu et al. 2025), among others. These genomic resources have facilitated the development of dense genetic maps and the identification of quantitative trait loci (QTLs) associated with agronomic and commercial relevant traits (Veerappan et al. 2021; Gracia et al. 2025; Itam et al. 2025; Mas-Gómez et al. 2025; Nicolás-Almansa et al. 2025). In apricot, the construction of genetic maps using segregating populations has led to the detection of QTLs controlling fruit quality attributes, including skin color, firmness, soluble solids content, and ripening time (Socquet-Juglard et al. 2013; García-Gómez et al. 2019). These studies have provided a foundation for the development of molecular markers tightly linked to candidate genes (Bianchi et al. 2015; García-Gómez et al. 2020), enabling more precise and efficient selection strategies.

Parallel to these advances, new types of molecular markers have been developed and applied across *Prunus* species. Simple sequence repeats (SSRs) remain widely used due to their high polymorphism, codominant inheritance, and reproducibility, especially when located within or near genes of interest (Wünsch and Hormaza 2002). Single nucleotide polymorphisms (SNPs), on the other hand, have gained prominence due to their abundance throughout the genome and compatibility with high-throughput genotyping platforms. Techniques such as genotyping-by-sequencing (GBS) and high-resolution melting (HRM) analysis have enabled the rapid identification and validation of SNP markers associated with key traits in fruit crops (Guajardo et al. 2015; Passaro et al. 2017; Carrasco et al. 2018; Salazar et al. 2019, 2026a; Chou et al. 2020).

Fruit skin color is one of the most important quality traits in apricot, as greater or lesser visual appeal strongly influences consumer preference and market trends. In general, yellow-skinned cultivars tend to exhibit excellent organoleptic properties; however, they are often associated with less favorable postharvest performance. In contrast, current market demand increasingly favors more intensely orange fruits, as they are considered more visually attractive. Consequently, apricot breeding programs face the challenge of developing varieties that are more appealing to consumers while also offering superior sensory quality (Gatti et al. 2009). In this regard, if the goal is to improve the flavor quality of orange-colored hybrids in a potential cross between yellow- and orange-skinned parents, the availability of reliable molecular markers associated with skin color would allow breeders to select orange-skinned seedlings with a greater likelihood of enhanced flavor quality, due to the inheritance of the yellow parent, which is more commonly associated with higher sugar content and aroma. Building on this premise, the present study integrates QTL mapping, candidate gene analysis, and marker development to identify robust molecular markers linked to apricot skin color, providing breeders with practical tools to select orange-skinned progeny while preserving the contribution of high-quality, yellow-skinned germplasm.

## Results

### Characterization of SNP—skin color alleles using HRM

**Fig. 1:**
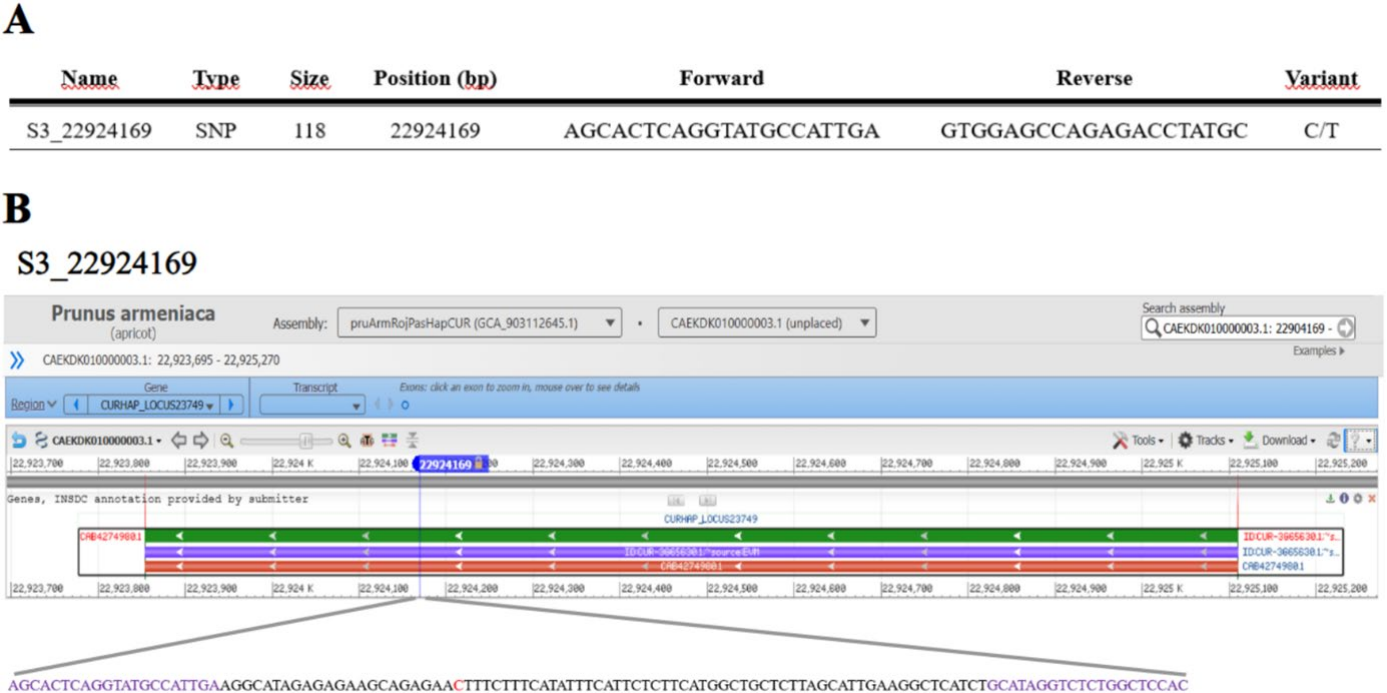
(A) Characteristics of the SNP marker S3_22924169, including marker type, amplicon size, genomic position, primer sequences, and allelic variation (C/T). (B) Genomic localization of SNP S3_22924169 within the *Prunus armeniaca* genome, showing its position in the candidate AP2/ERF gene and the primer-binding regions used for HRM analysis

Apricot skin color has been strongly associated with the distal region of LG3, differentiating yellow and orange phenotypes in two apricot populations (Salazar et al. 2026a). The major QTLs were mapped between 22 and 23 Mbp and were primarily linked to carotenoid biosynthesis. Accordingly, the most significant SNPs, explaining the highest proportion of phenotypic variance, were selected for allelic characterization using HRM; notably, SNP S3_22924169 (Fig. 1). This SNP is located in an intragenic region of the gene encoding an AP2/ERF domain–containing protein (Prupe.3G263000) and showed a highly significant association with fruit skin color in the ‘Goldrich’ × ‘Currot’ population (p-value = 8.27 × 10⁻^22^; R^2^ = 0.39) (Salazar et al. 2026a).

HRM assays were conducted on 40 selected genotypes from the ‘Goldrich’ × ‘Currot’ population, representing yellow- and orange-skinned fruits, to validate the SNP, showing a clear association between allelic variants and fruit skin color (Table S1). The C/T genotype was consistently associated with orange skin color (h° < 77), whereas the C/C genotype corresponded to yellow skin color (h° > 83) (Table 1).

**Table 1:** HRM-based genotyping of SNP S3_22924169 and its association with fruit skin color phenotypes in the ‘Goldrich’ × ‘Currot’ population

**ID**	**GxC**	**Ripening**	**S3_22924169**	**Skin color (h**°**)**	**Phenotype**^**a**^
P1	GxC_10-16	06/11/2025	C/T	72.3 ± 0.7	Orange
P2	GxC_5-17	06/11/2025	C/T	73.4 ± 0.4	Orange
P3	GxC_7-10	30/05/2025	C/T	73.5 ± 1.4	Orange
P4	GxC_5-9	06/06/2025	C/T	73.8 ± 0.3	Orange
P5	GxC_7-11	06/11/2025	C/T	74.3 ± 0.5	Orange
P6	GxC_7-5	06/11/2025	C/T	74.3 ± 0.1	Orange
P7	GxC_5-13	06/06/2025	C/T	74.7 ± 0.5	Orange
P8	GxC_11-14	06/11/2025	C/T	75.1 ± 0.3	Orange
P9	GxC_11-1	06/11/2025	C/T	75.3 ± 0.5	Orange
P10	GxC_4-4	06/06/2025	C/T	75.4 ± 0.6	Orange
P11	GxC_7-14	06/06/2025	C/T	75.6 ± 0.4	Orange
P12	GxC_4-17	27/05/2025	C/T	75.9 ± 3.2	Orange
P13	GxC_3-7	06/11/2025	C/T	76.0 ± 0.3	Orange
P14	GxC_11-3	06/11/2025	C/T	76.0 ± 0.3	Orange
P15	GxC_7-16	06/06/2025	C/T	76.0 ± 0.3	Orange
P16	GxC_6-15	06/06/2025	C/T	76.1 ± 1.1	Orange
P17	GxC_6-18	30/05/2025	C/T	76.1 ± 1.7	Orange
P18	GxC_8-19	30/05/2025	C/T	76.4 ± 2.1	Orange
P19	GxC_2-6	30/05/2025	C/T	76.7 ± 1.8	Orange
P20	GxC_1-9	30/05/2025	C/T	76.9 ± 1.4	Orange
P21	GxC_9-19	06/06/2025	C/T	76.9 ± 0.2	Orange
P22	GxC_2-8	06/06/2025	C/C	85.1 ± 0.4	Yellow
P23	GxC_5-7	06/06/2025	C/C	85.5 ± 0.5	Yellow
P24	GxC_2-7	06/06/2025	C/C	86.3 ± 0.3	Yellow
P25	GxC_7-15	06/06/2025	C/C	86.5 ± 1.5	Yellow
P26	GxC_8-2	30/05/2025	C/C	86.6 ± 1.6	Yellow
P27	GxC_2-2	06/06/2025	C/C	86.8 ± 0.8	Yellow
P28	GxC_10-3	30/05/2025	C/C	86.9 ± 0.4	Yellow
P29	GxC_11-10	06/06/2025	C/C	87.0 ± 0.5	Yellow
P30	GxC_11-19	06/06/2025	C/C	87.9 ± 0.2	Yellow
P31	GxC_9-16	06/06/2025	C/C	88.0 ± 0.3	Yellow
P32	GxC_2-10	06/06/2025	C/C	88.1 ± 0.2	Yellow
P33	GxC_3-10	06/06/2025	C/C	88.1 ± 0.4	Yellow
P34	GxC_1-2	30/05/2025	C/C	88.9 ± 1.4	Yellow
P35	GxC_7-17	30/05/2025	C/C	89.0 ± 1.3	Yellow
P36	GxC_4-6	06/11/2025	C/C	89.3 ± 1.1	Yellow
P37	GxC_6-10	06/06/2025	C/C	90.2 ± 0.6	Yellow
P38	GxC_4-11	30/05/2025	C/C	90.3 ± 0.7	Yellow
P39	GxC_8-8	30/05/2025	C/C	90.9 ± 1.9	Yellow
P40	GxC_2-11	06/06/2025	C/C	92.4 ± 0.8	Yellow

^a^“Phenotype” corresponds to hue-based classification: Orange (h° < 77), Light Orange (77 < h° < 83), or Yellow (h° > 83)

**Fig. 2:**
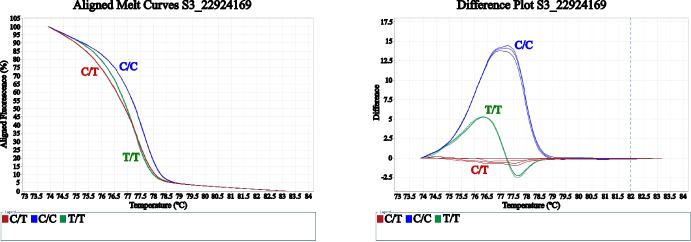
High-resolution melting (HRM) analysis of SNP S3_22924169 showing aligned normalized melting curves (left) and difference plots (right), allowing discrimination among the three genotypic classes: C/C (blue), C/T (red), and T/T (green)

Once the skin‑color molecular marker for HRM was shown to work with 100% efficiency in seedlings with extremely orange and yellow skin, we decided to validate this primer in a variety collection. When the SNP molecular marker was applied to the panel of 57 apricot cultivars, all three possible genotypic classes were clearly distinguished by HRM melting curves, both in normalized fluorescence loss and difference plots: the C/C homozygote, the C/T heterozygote, and the newly identified T/T homozygote (Fig. 2).

Skin color analysis across the evaluated cultivars revealed that orange-fruited cultivars such as ‘Cebas Red’, ‘Flopria’, ‘Cebas57’, ‘Magic Cot’, ‘Tsunami’, and ‘Estrella’ carried the T/T genotype. This observation supports an association between the presence of the thymine base and orange fruit skin color in apricot, as most cultivars harboring the C/T genotype also exhibited this coloration, whereas yellow skin color was predominantly associated with the C/C genotype (Table 2). Although the correlation between phenotype and genotype within the cultivar set was high (89.5% unweighted; 93.5% weighted) using this SNP-based molecular marker, additional genetic factors are likely involved in the control of this trait. Consequently, although this HRM‑based approach provides very high efficiency, we decided to search for a more robust molecular marker to facilitate its potential application as a marker‑assisted selection tool in a breeding program. Therefore, the development of SSR markers was further pursued, with particular focus on the candidate gene AP2/ERF domain–containing protein.

**Table 2:** Application of SNP S3_22924169 through HRM analysis and its association with fruit skin color phenotypes in 57 apricot cultivars

**ID**	**Cultivar**	**Ripening**	**S3_22924169**	**Skin color (h**°**)**	**Phenotype**^**a**^
C1	Totem	22/05/2025	C/T	51.8 ± 6.6	Orange
C2	Cheyenne	06/06/2025	C/T	63.0 ± 1.3	Orange
C3	Fuego	27/05/2025	C/T	63.5 ± 5.7	Orange
C4	Playa Cot	12/06/2025	C/T	64.4 ± 0.9	Orange
C5	Pricia	30/05/2025	C/T	65.5 ± 2.6	Orange
C6	Cebas Red	12/05/2025	T/T	65.9 ± 2.0	Orange
C7	Sunglo	03/07/2025	C/T	66.0 ± 0.8	Orange
C8	Micaelo	16/06/2025	C/T	66.5 ± 1.1	Orange
C9	Orange Ruby	12/06/2025	C/T	66.9 ± 0.5	Orange
C10	Flopria	06/06/2025	T/T	66.9 ± 1.2	Orange
C11	Toñi	06/06/2025	C/T	67.3 ± 1.0	Orange
C12	L3-11	22/05/2025	C/T	67.5 ± 1.2	Orange
C13	Colorado	16/05/2025	C/T	67.9 ± 2.1	Orange
C14	Rosa	06/06/2025	C/T	68.0 ± 0.4	Orange
C15	Palsteyn	06/06/2025	C/C	68.3 ± 1.1	Orange
C16	Bergarouge	03/07/2025	C/T	68.9 ± 1.1	Orange
C17	Goldrich	11/06/2025	C/T	68.9 ± 1.0	Orange
C18	Monster Cot	12/06/2025	C/T	69.2 ± 0.6	Orange
C19	Selene	06/06/2025	C/T	69.3 ± 1.1	Orange
C20	Tardorange	19/06/2025	C/T	69.3 ± 1.6	Orange
C21	Lady Cot	16/06/2025	C/T	69.5 ± 1.1	Orange
C22	Harogem	19/06/2025	C/T	69.5 ± 2.1	Orange
C23	Rubisco	30/05/2025	C/T	69.8 ± 2.2	Orange
C24	Maya Cot	20/05/2025	C/T	69.8 ± 2.1	Orange
C25	Cebas57	20/05/2025	T/T	70.0 ± 0.7	Orange
C26	Mirlo Blanco	20/05/2025	C/T	70.1 ± 1.5	Orange
C27	Magic Cot	27/05/2025	T/T	70.3 ± 2.8	Orange
C28	Orange Red	11/06/2025	C/T	70.3 ± 0.4	Orange
C29	11_1	19/06/2025	C/T	71.1 ± 0.5	Orange
C30	Tsunami	30/05/2025	T/T	71.1 ± 0.8	Orange
C31	Murciana	12/06/2025	C/T	71.2 ± 1.1	Orange
C32	Lilly Cot	22/05/2025	C/T	71.9 ± 0.6	Orange
C33	Sublime	30/05/2025	C/T	72.6 ± 2.8	Orange
C34	Valorange	06/06/2025	C/T	72.6 ± 1.0	Orange
C35	Mirlo Naranja	16/05/2025	C/T	72.8 ± 2.2	Orange
C36	Maravilla	06/06/2025	C/T	72.8 ± 0.3	Orange
C37	Deseo	19/06/2025	C/T	72.9 ± 0.2	Orange
C38	Estrella	30/05/2025	T/T	73.1 ± 2.4	Orange
C39	Mirlo Rojo	27/05/2025	C/T	73.5 ± 2.2	Orange
C40	Micado	20/05/2025	C/T	73.9 ± 2.2	Orange
C41	Tyrinthos	27/05/2025	C/C	74.5 ± 1.1	Orange
C42	Lito	19/06/2025	C/C	75.7 ± 2.4	Light Orange
C43	San Castrese	12/06/2025	C/C	78.1 ± 0.2	Light Orange
C44	Bebeco	12/06/2025	C/C	78.5 ± 0.2	Light Orange
C45	Rojo Pasión	30/05/2025	C/T	79.4 ± 1.7	Light Orange
C46	Bergeron	27/06/2025	C/C	79.5 ± 1.8	Light Orange
C47	906–12	30/05/2025	C/T	79.7 ± 2.2	Light Orange
C48	Helena	03/07/2025	C/C	83.8 ± 0.8	Yellow
C49	Canino	30/05/2025	C/C	85.3 ± 1.6	Yellow
C50	Real Fino	19/06/2025	C/C	87.4 ± 2.8	Yellow
C51	Mauricio	29/05/2025	C/C	87.6 ± 1.4	Yellow
C52	Dorada	03/07/2025	C/C	88.9 ± 0.8	Yellow
C53	Capricho	12/06/2025	C/C	90.0 ± 0.7	Yellow
C54	Pepito del Rubio	12/06/2025	C/C	90.0 ± 0.2	Yellow
C55	Moniqui	16/06/2025	C/C	90.2 ± 0.7	Yellow
C56	Currot	20/05/2025	C/C	90.6 ± 3.0	Yellow
C57	Guillermos	12/06/2025	C/C	90.9 ± 0.4	Yellow

^a^“Phenotype” corresponds to hue-based classification: Orange (h° < 77), Light Orange (77 < h° < 83), or Yellow (h° > 83)

### Characterization of SSR—Skin color alleles using ABI sequencing

Among the developed markers, Col1, located within the AP2/ERF domain–containing protein gene (Prupe.3G263000) that harbors the previously described SNP, exhibited the highest classification efficiency, reaching 93.9% when calculated as weighted efficiency and 96.4% as unweighted efficiency (Table S2). The genotype ‘Maya Cot’ (C24, Table 3), which showed an “X/X” allelic profile, was excluded from the efficiency calculation, as this result indicates that no PCR product was detected, most likely due to a technical failure during amplification. This result indicates a strong and reliable association between SSR allelic variation and apricot skin color. Four distinct SSR allele sizes were detected (108, 111, 114, and 120 bp), of which the 108 bp allele was predominantly associated with yellow-skinned cultivars, whereas the 111, 114, and 120 bp alleles were mainly associated with orange skin color. The few cultivars that did not correlate with the expected phenotype were ‘Bebeco’ and ‘Helena’, both displaying hue values within the transitional range of the color spectrum (Table 3). In these cases, harvest timing is considered a critical factor influencing phenotypic assessment, as slight differences in ripening stage can affect skin color measurements and potentially lead to phenotyping inaccuracies.

In contrast, the remaining SSR markers (Col2, Col3, and Col4) did not show a clear and consistent pattern linking allelic profiles with skin color classes. Markers located within genes encoding a ζ-carotene desaturase, MYB30, and MYB21-related transcription factors displayed lower efficiencies, ranging from 60.0% to 83.9%, and were associated with pronounced phenotypic misclassifications.

These findings highlight the AP2/ERF-based SSR marker (Col1) as the most robust and informative marker for apricot skin color discrimination among those evaluated. For this reason, we further aimed to investigate the genetic architecture underlying this gene to better understand its contribution to color variation in apricot.

**Table 3:**
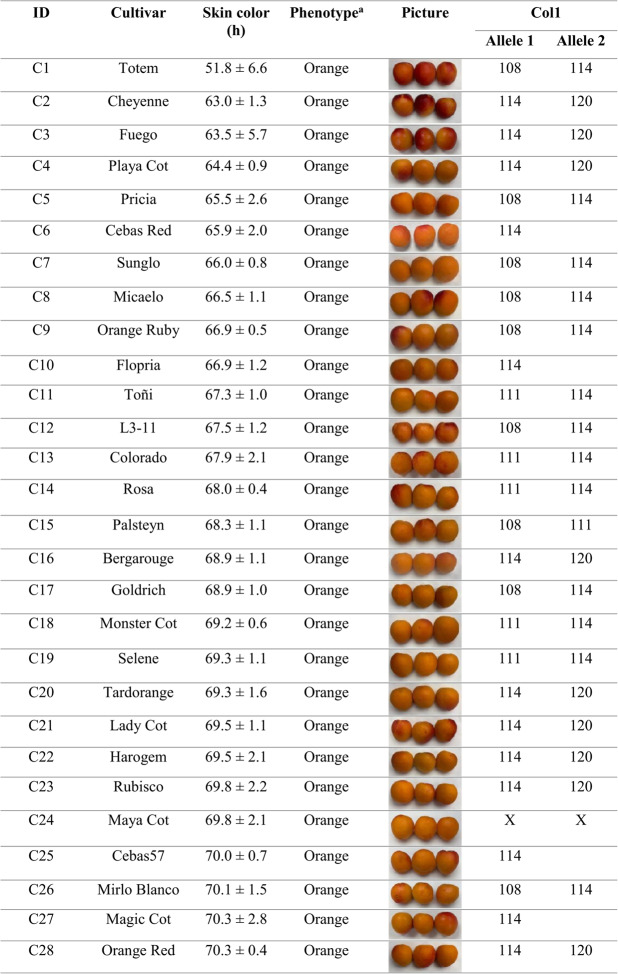

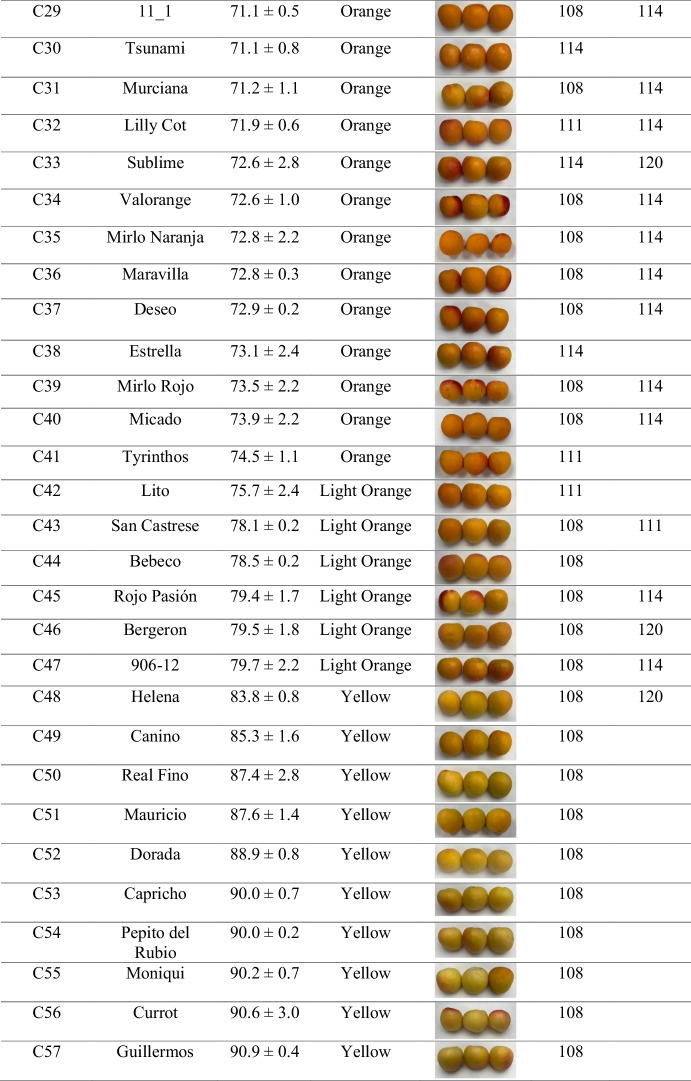
Genotyping data of Col1 through SSR analysis and its association with fruit skin color phenotypes in 57 apricot cultivars

^a^“Phenotype” corresponds to hue-based classification: Orange (h° < 77), Light Orange (77 < h° < 83), or Yellow (h° > 83).

### Allelic reconstruction of the AP2/ERF gene

**Fig. 3:**
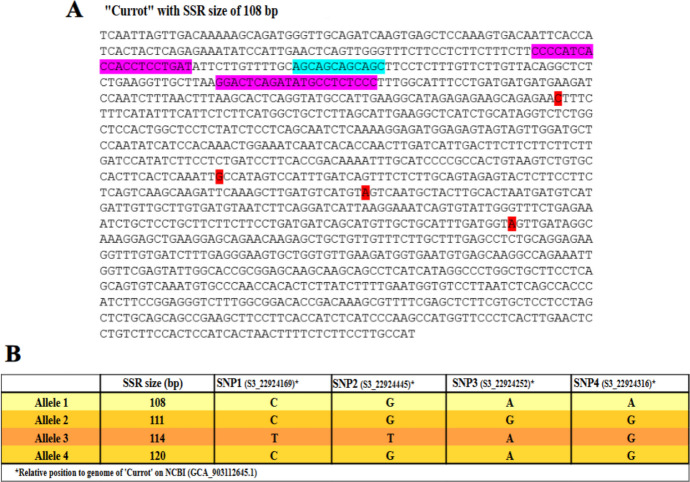
Allelic structure of the AP2/ERF gene. (A) Nucleotide sequence of the reference allele showing the intragenic AGC-based SSR motif and the positions of the four SNPs identified within the gene (primers in purple). (B) Summary of the four reconstructed alleles defined by SSR length variation (108, 111, 114, and 120 bp) and their corresponding SNP haplotypes, including SNP1 used for HRM analysis in this study. The orange color intensity indicates the linkage between alleles and carotenoid content

Reconstruction of AP2/ERF gene alleles was performed using genomic sequences from publicly available apricot genomes and SRA datasets, as detailed in (Table S3). Sequence analysis revealed the presence of an intragenic SSR composed of an AGC trinucleotide repeat motif, together with four SNPs distributed along the gene (Fig. 3). The SSR showed four distinct allelic sizes: 108, 111, 114, and 120 bp, reflecting variation in the number of AGC repeats. Among the identified SNPs, SNP1 (SNP S3_22924169) corresponds to the variant previously detected by GBS and validated by HRM analysis in this study, while SNP2 (SNP S3_22924445), SNP3 (SNP S3_22924252), and SNP4 (SNP S3_22924316) were identified through sequence alignment–based comparisons of the target gene across different cultivars.

The combined SSR length polymorphism and SNP variation allowed the definition of four distinct AP2/ERF alleles (Table S4). Allele 1 (SSR108) was characterized by the SNP combination C–G–A–A, Allele 2 (SSR111) by C–G–G–G, Allele 3 (SSR114) by T–T–A–G, and Allele 4 (SSR120) by C–G–A–G.

### Protein reconstruction of the AP2/ERF gene

**Fig. 4:**
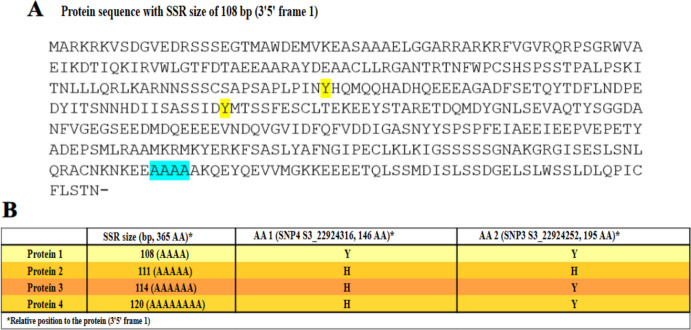
Reconstruction of AP2/ERF protein variants derived from different alleles. (A) Amino acid sequence of the reference protein showing the polyalanine tract encoded by the AGC-based SSR and the amino acid positions affected by non-synonymous SNPs. (B) Summary of the four reconstructed protein variants indicating SSR length–dependent polyalanine expansion and amino acid substitutions at positions affected by SNP3 and SNP4, where guanine (G) encodes histidine (H) and adenine (A) encodes tyrosine (Y). The orange color intensity indicates the linkage between alleles and carotenoid content

Protein sequences encoded by the different allelic variants of the AP2/ERF gene were reconstructed to evaluate the functional consequences of SSR and SNP polymorphisms (Fig. 4). Translation of the reconstructed allelic sequences revealed that the intragenic SSR, based on an AGC trinucleotide repeat, directly affects the length of a polyalanine tract within the protein. Specifically, variation in SSR length (108, 111, 114, and 120 bp) resulted in progressive expansion of the alanine stretch, ranging from four to eight consecutive alanine residues, without altering the overall protein reading frame. In contrast to the SSR, only two of the four identified SNPs resulted in amino acid substitutions in the protein sequence. These corresponded to SNP3 and SNP4, which affected amino acid positions 195 and 146, respectively (relative to the 3′–5′ frame 1). In both cases, the nucleotide present at the SNP position determined the encoded residue, with guanine (G) specifying histidine (H) and adenine (A) specifying tyrosine (Y). The remaining SNPs were synonymous and did not lead to changes in the amino acid sequence.

The combination of SSR-driven polyalanine length variation and SNP-induced amino acid substitutions allowed the definition of four distinct AP2/ERF protein variants (Table S5). Protein 1 (SSR108) carried a shorter polyalanine tract and encoded tyrosine residues at both variable amino acid positions. Protein 2 (SSR111) exhibited an intermediate alanine expansion and histidine substitutions at both positions. Protein 3 (SSR114) combined a longer alanine tract with a histidine-to-tyrosine configuration, whereas Protein 4 (SSR120) displayed the longest polyalanine stretch together with a mixed histidine and tyrosine pattern.

### Characterization of SNP4 as a candidate marker for skin color using HRM

To further explore the functional relevance of non-synonymous variation within the AP2/ERF gene, a candidate molecular marker based on SNP4 (S3_22924316) was developed and validated using HRM analysis. This SNP, identified through sequence alignment, results in an amino acid substitution and was therefore considered a strong candidate for improving genotype–phenotype discrimination. Primer pairs amplified a 109 bp fragment, achieving 96.4% unweighted efficiency and 93.9% weighted efficiency (Table 4).

**Table 4:** Primer sequences and efficiency parameters of the SNP4 HRM marker for apricot skin color

ID	Apricot color primers		Size (bp)	Efficiency (%)	Weighted efficiency (%)
SNP4 S3_22924316	TCCTGCTTCTTCTTCCTGATGA	Forward	109	96.4	93.9
	GCTCAAAGCAAGAAACAACAGC	Reverse			

**Fig. 5:**
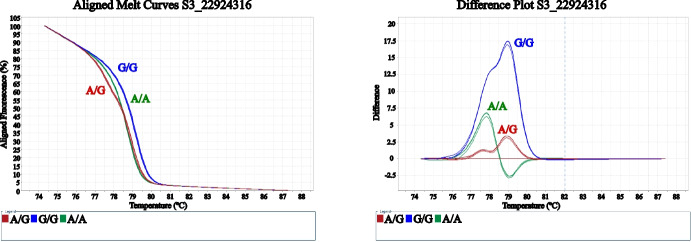
High-resolution melting (HRM) analysis of SNP4 S3_22924316 showing aligned normalized melting curves (left) and difference plots (right), allowing discrimination among the three genotypic classes: G/G (blue), A/G (red), and A/A (green)

HRM analysis of SNP4 generated clearly distinguishable melting profiles, enabling the identification of the three genotypic classes (G/G, A/G, and A/A). Both normalized melting curves and difference plots showed well-defined clustering patterns, confirming the robustness and reproducibility of this marker for genotype discrimination (Fig. 5). The A/A genotype was associated with yellow skin color, whereas G/G and A/G genotypes were associated with orange skin color, although without discriminating among different orange tonalities.

Genotyping results obtained with SNP4 showed a strong concordance with those previously observed for the SSR marker, confirming the consistency of the genetic signal associated with skin color. The same cultivars, ‘Bebeco’ and ‘Helena’, were identified as misclassified, as previously discussed. These discrepancies are likely attributable to phenotyping inaccuracies, particularly related to slight differences in fruit ripening stage that may affect color measurement (Table 5).

**Table 5:**
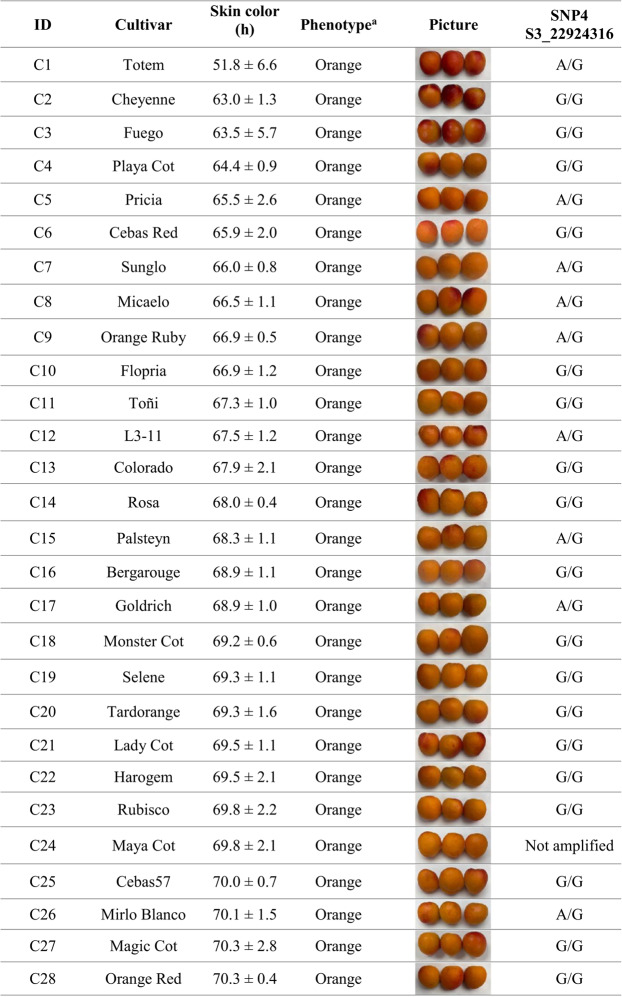

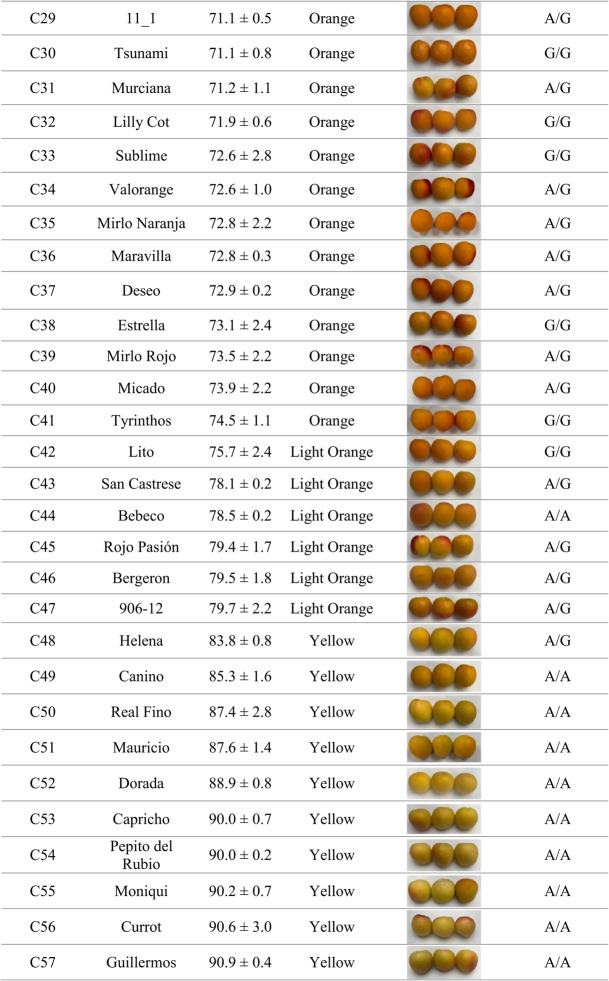
Genotyping data of SNP4 through HRM analysis and its association with fruit skin color phenotypes in 57 apricot cultivars

^a^“Phenotype” corresponds to hue-based classification: Orange (h° < 77), Light Orange (77 < h° < 83), or Yellow (h° > 83)

## Discussion

Fruit skin color in apricot is a complex trait largely determined by the accumulation and composition of carotenoids, which are synthesized through a highly conserved biosynthetic pathway in plants (Lado et al. 2016). In *Prunus* species, carotenoid biosynthesis begins with the condensation of geranylgeranyl diphosphate into phytoene, a reaction catalyzed by phytoene synthase (PSY), which is widely recognized as a key rate-limiting enzyme in the pathway. Subsequent desaturation and isomerization steps, mediated by enzymes such as phytoene desaturase and ζ-carotene desaturase, lead to the production of colored carotenoids including β-carotene and xanthophylls (Yin et al. 2021). The regulation of this pathway occurs at both transcriptional and post-transcriptional levels and is tightly coordinated with fruit development and ripening (Yan et al. 2020).

Transcription factors play a central role in modulating carotenoid accumulation by regulating the expression of structural genes within this pathway (Jiang et al. 2019). Among them, members of the AP2/ERF superfamily have been repeatedly implicated in fruit development and ripening across diverse species, including *Prunus mume* (Du et al. 2013), *Prunus sibirica* (Zhang et al. 2024), *Prunus persica* (Zhang et al. 2012), and *Prunus avium* (Wang et al. 2024). AP2/ERF proteins are known to integrate hormonal signals, particularly ethylene, and to act either as transcriptional activators or repressors depending on their structure and interacting partners (Feng et al. 2020; Zhai et al. 2022). In climacteric and non-climacteric fruits alike, ERF transcription factors have been shown to directly regulate genes involved in pigment biosynthesis influencing fruit coloration (Xie et al. 2016).

In this study, we identified and characterized an AP2/ERF domain–containing gene located within a major apricot skin color QTL on chromosome 3. Both SNP- and SSR-based markers developed within this gene showed strong associations with fruit skin color, supporting its functional relevance. The high efficiency of the AP2/ERF-based SSR marker (93.9%) and the robust correlation observed with the SNP marker further reinforce the central role of this gene in determining color variation in apricot. These findings are consistent with previous reports in apricot and apple, where AP2/ERF transcription factors have been linked to carotenoid regulation and fruit quality traits (Zhang et al. 2019; Dang et al. 2021).

**Fig. 6:**
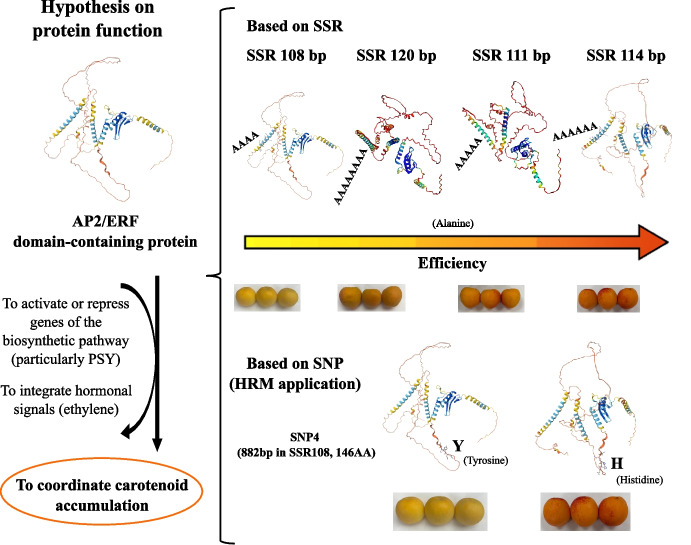
Proposed hypothesis for the functional role of allelic variation in the AP2/ERF domain–containing protein in apricot fruit skin color regulation

A key contribution of this work is the reconstruction of allelic and protein variants of the AP2/ERF gene, which allowed us to propose a mechanistic hypothesis explaining how genetic variation at this locus may translate into phenotypic differences in fruit color (Fig. 6). Our results demonstrate that allelic diversity in the AP2/ERF gene arises through two complementary mechanisms: variation in the length of an intragenic AGC-based SSR, which modulates the size of a polyalanine tract in the encoded protein, and non-synonymous SNPs that lead to amino acid substitutions. Importantly, only two of the four identified SNPs (SNP3 and SNP4) resulted in amino acid changes, replacing histidine with tyrosine depending on the nucleotide state (G or A). Together, these polymorphisms generate distinct protein isoforms that likely differ in stability, folding, or transcriptional activity.

Polyalanine tracts are known to influence protein function by affecting protein–protein interactions, subcellular localization, and transcriptional regulatory capacity (Kottenhagen et al. 2012; Mier et al. 2022). In the context of AP2/ERF proteins, expansion of alanine residues may alter the ability of the protein to interact with co-regulators or to bind DNA efficiently. Our results suggest that this effect is not linear, but instead follows an optimal-length model. Specifically, the shortest SSR allele (SSR108), encoding a polyalanine tract of four residues, and the longest allele (SSR120), encoding eight alanines, appear to be less functionally efficient. In contrast, the intermediate SSR114 allele, corresponding to a tract of six alanine residues, is associated with enhanced regulatory efficiency. This intermediate length may provide an optimal balance between structural stability and conformational flexibility, facilitating effective DNA binding and interaction with co-regulatory proteins.

The amino acid substitutions introduced by SNP4 further contribute to protein functional diversity. Histidine and tyrosine differ substantially in their chemical properties, with histidine playing a potential role in pH-sensitive interactions and tyrosine often involved in phosphorylation or aromatic stacking (Ingle 2011; Song et al. 2022). Substitutions at key positions within the AP2/ERF protein could therefore influence transcriptional activity, signal integration, or protein turnover. Our reconstructed sequence analysis revealed a strong association between the histidine-containing variants and orange skin color, whereas tyrosine-containing variants were predominantly associated with yellow fruits. This observation supports the hypothesis that these amino acid changes may affect the regulatory capacity of AP2/ERF with respect to carotenoid accumulation; therefore, SNP4 emerges as an optimal candidate for the development of a molecular marker using HRM analysis.

Taken together, while functional validation experiments will be necessary to confirm this hypothesis, the strong genotype–phenotype associations observed in this study provide compelling indirect evidence for a causal relationship.

An additional aspect of particular relevance is the observed correlation between fruit skin color and flesh color in apricot (Ruiz et al. 2005). Carotenoid accumulation in the skin is often accompanied by parallel changes in the flesh, reflecting shared regulatory mechanisms and overlapping biosynthetic pathways. Therefore, the AP2/ERF-based markers described here may also serve as indirect markers for flesh color, further increasing their utility in breeding programs. The ability to predict both skin and flesh color at the seedling stage represents a valuable advantage, especially for breeding programs targeting specific market segments.

Within the genus *Prunus*, molecular markers associated with fruit color have been more extensively developed for anthocyanin-based coloration, particularly in plum (Fiol et al. 2021) and peach (Guo et al. 2023). In these species, red or purple coloration is largely driven by anthocyanin accumulation, and several MYB and bHLH transcription factors have been identified as key regulators (Fiol et al. 2019). In apricot, however, carotenoids remain the primary contributors to skin color, although an increasing number of modern cultivars display red blush or fully red skin due to localized or extensive anthocyanin accumulation (Xi et al. 2019). The growing presence of red-skinned apricots in the market reflects changing consumer preferences and represents a new challenge and opportunity for breeders. As red coloration becomes more prominent and, in some cases, covers the entire fruit surface, there is a clear need to identify molecular markers associated with anthocyanin biosynthesis and regulation in apricot. Future studies should therefore aim to integrate carotenoid- and anthocyanin-related markers to enable a more comprehensive genetic dissection of fruit color. The AP2/ERF marker developed in this work provides a foundation upon which additional markers targeting anthocyanin pathways can be built.

From an applied perspective, the molecular marker developed in this study represents a practical tool for apricot breeding programs. Its high efficiency and robustness make it particularly suitable for early selection of seedlings with orange or yellow skin color. This is especially relevant in breeding schemes where one of the parental cultivars has yellow skin but superior organoleptic properties, such as higher sweetness, aroma, or texture. By enabling early discrimination between orange- and yellow-skinned progeny, breeders can more efficiently combine desirable quality traits with market-preferred coloration.

## Methods

### Plant material and experimental design

The plant material evaluated in 2025 comprised a set of 57 commercial cultivars and 40 genotypes from the F1 population derived from the cross ‘Goldrich’ × ‘Currot’. All plant materials were grown in the CEBAS-CSIC experimental orchard located in Cieza–Calasparra, Murcia, Spain (37° N, 1° W; 450 m a.s.l.). Three fruits per genotype were collected for skin-color assessment. Fruit harvest was performed according to external color transition and field firmness criteria. For each genotype, genomic DNA was extracted from freeze-dried young leaves using a standard CTAB protocol (Maguire et al. 1994). DNA concentrations were measured with a UV–visible microvolume spectrophotometer called ThermoScientific™ NanoDrop™ (Madrid, Spain) and subsequently diluted to 30 ng/µL.

### Phenotypic characterization of fruit color

**Fig. 7:**
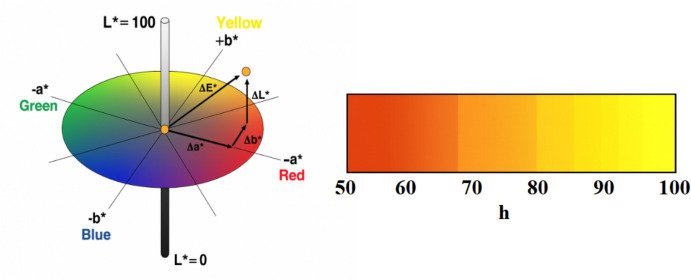
Left: representation of the CIELAB color space showing the L*, a*, and b* axes and the hue angle (h°). Right: magnified view of the hue angle range relevant to this study, illustrating the color gradient used to classify apricot skin color from orange to yellow

Fruit color was assessed using a Minolta CR-400 colorimeter (Minolta, Ramsey, NJ, USA). For each genotype, two measurements per fruit were recorded: one on each opposite side of the fruit skin, resulting in a total of six measurements. Special care was taken to ensure that measurements corresponded exclusively to the ground color of the fruit peel, avoiding any interference from the overcolor (blush). Instrument calibration was performed using a white porcelain reference plate. Color was quantified in the CIELAB space by determining L* (lightness), a* (red–green), and b* (yellow–blue) (Fig. 7). The hue angle (hº = arctan [b*/a*]) was also calculated following (Brown and Walker 1990), and values were categorized into hue-based classes: orange (h° < 77), light orange (77 < h° < 83), and yellow (h° > 83). According to the EU standards (Guidelines for the Conduct of Tests for Distinctness, Uniformity and Stability. Apricot (*Prunus armeniaca* L.), TG/70/5) (UPOV 2021), seven categories of apricot peel base color are defined (not visible, white, yellowish, yellow green, light orange, medium orange, and dark orange). However, in the present study these categories were grouped into three classes to provide a more robust approximation in the presence of polymorphism-related variability: not visible, white, yellowish, and yellow green were grouped as “yellow”; light orange was maintained as “light orange”; and medium orange together with dark orange were grouped as “orange.”

### SNP-based marker development for the AP2/ERF gene using HRM analysis

The sequence of the SNP marker S3_22924169 selected based on its high statistical significance reported in previous QTL studies on fruit quality traits in apricot (Salazar et al. 2026a), was used to identify the corresponding candidate gene by using the Phytozome 14 platform (https://phytozome-next.jgi.doe.gov/). Primer pairs for HRM analysis were designed with Primer3Plus (Untergasser et al. 2007). The primer design for SNP S3_22924169 was optimized to generate an amplicon of approximately 100 bp. This fragment size is considered optimal for detecting sequence variation by HRM analysis, and the presence of a C/T polymorphism further enhances discrimination based on melting temperature differences (Simko 2016). HRM was performed on the ‘Goldrich’ × ‘Currot’ population, as well as on 57 apricot cultivars to assess its reproducibility and applicability as a molecular marker. Additionally, a candidate marker for skin color was also developed through HRM analysis based on SNP4 (S3_22924316), following its identification during the analysis of gene sequences (Table 4).

HRM assays were conducted using a StepOnePlus™ Real-Time PCR System (Applied Biosystems). Each reaction was carried out in a total volume of 10 µL, consisting of 5 µL of 2 × HRM MeltDoctor™ Master Mix (Applied Biosystems™), 0.25 µL of each primer (10 µM), and 1 µL of genomic DNA (30 ng/µL). The amplification protocol included an initial denaturation step at 95 °C for 10 min, followed by 40 cycles of 95 °C for 15 s and 60 °C for 1 min. Melting curve analysis was performed with an initial step at 95 °C for 15 s and 60 °C for 1 min, followed by a gradual temperature increase of 0.3 °C increments up to 95 °C, with a hold of 0.15 s at each step. HRM data were analyzed using the HRM Plug-in for DA3 Software (Applied Biosystems™). Melting profiles were normalized according to the manufacturer’s instructions and visualized as normalized fluorescence versus temperature curves and derivative plots (-dF/dT).

### SSR marker development for color-related genes

For the development of microsatellite markers, the genomic region spanning 22 to 25 Mb on chromosome 3, which contains the major apricot color QTL, was examined (Salazar et al. 2026a). Using the Phytozome platform and BLAST searches against the *Prunus persica* v2.1 genome (Goodstein et al. 2012), four color-related genes containing SSR repeats were identified within this interval, as summarized in Table 6. This approach was adopted because *Prunus persica* is the only species within the genus *Prunus* with a well-curated functional genome annotation, allowing reliable inference of candidate genes through orthologous relationships, given the limited annotation currently available for apricot genomes. Primer pairs for each locus were designed using Primer3Plus, and forward SSR primers were fluorescently labeled with HEX dyes.

**Table 6:** Characteristics of SSR markers developed within candidate genes located in the major apricot skin color QTL region on chromosome 3

ID	NCBI ("Currot" genome)	Gene (*Prunus persica* v2.1 from Phytozome)	Apricot color primers		Size (bp)
Col1	CAEKDK010000003.1: 22,923,854–22,925,110	Prupe.3G263000: (1 of 1) PTHR31194:SF197—AP2_ERF DOMAIN-CONTAINING PROTEIN	Hex-CCCCATCACCACCTCCTGAT	Forward	114
			GGGAGAGGCATATCTGAGTCC	Reverse	
Col2	CAEKDK010000003.1: 23,048,365–23049155	Prupe.3G264800: (1 of 3) 1.3.5.6—9,9'-di-cis-zeta-carotene desaturase	Hex-CGTTTCAATCTGTCCCTTGCA	Forward	169
		Prupe.3G264900: (1 of 11) PTHR36488:SF8—CASP-LIKE PROTEIN 1U1	CTCTCTCGACGCTGAGTGTC	Reverse	
Col3	CAEKDK010000003.1: 23,177,464–23,179,620	Prupe.3G268000: (1 of 1) PTHR10641:SF1379—TRANSCRIPTION FACTOR MYB30	Hex-TGTCATGCTTGCCAAGACTT	Forward	165
			GACATTGTACCCCACAGCTCA	Reverse	
Col4	CAEKDK010000003.1: 24,076,897–24080977	Prupe.3G286800: (1 of 1) PTHR45675:SF44—TRANSCRIPTION FACTOR MYB21-RELATED	Hex-CTTGTCTGTGTGATTGTGCGT	Forward	144
			ACCCCCAGCTACCCCATTAT	Reverse	

PCR amplifications were conducted in a final volume of 15 µL containing 1.5 µL of 10 × reaction buffer (Applied Biosystems, Foster City, CA, USA), 8.95 µL nuclease-free water, 1 µL MgCl₂ (25 mM), 0.3 µL dNTP mix (10 mM), 0.3 µL each of forward and reverse primers (10 mM), 0.15 µL Taq polymerase (500 U/µL; Fisher Molecular Biology, Rome, Italy), and 2.5 µL genomic DNA (30 ng/µL). Reactions were performed in a 2720 thermal cycler (Applied Biosystems) using the following program: initial denaturation at 95 °C for 2 min; 35 cycles of 95 °C for 40 s, 57 °C for 45 s, and 72 °C for 50 s; and a final extension at 72 °C for 5 min. For SSR genotyping, 1 µL of each PCR product was mixed with 9 µL of formamide and 0.2 µL of GeneScan 500 LIZ size standard (Applied Biosystems) and analyzed on an ABI PRISM 3730 DNA Analyzer. Fragment profiles were examined and scored using Peak Scanner v1.0 (Applied Biosystems).

### Allelic reconstruction of the AP2/ERF gene in apricot

The different allelic variants of the AP2/ERF gene were reconstructed using several available genomic resources: the ‘Currot’ and ‘Orange Red’ genomes deposited in NCBI (O’Leary et al. 2024); the ‘Marouch n14’ and ‘Stella’ genomes available in the GDR (Jung et al. 2019); and the ‘Lito’ genome (unpublished data). Additionally, SRA datasets from NCBI corresponding to cultivars such as ‘Bebeco’, ‘Bergeron’, ‘Canino’, ‘Moniqui’, ‘Murciana’, ‘Orange Red’, ‘Palsteyn’, and ‘San Castrese’ were incorporated (PRJNA292050 Prunus armeniaca var. armeniaca). Illumina resequencing reads of apricot cultivars retrieved from the NCBI SRA database were preliminarily screened by BLAST against the reference sequence of the AP2/ERF gene derived from the cultivar Currot. Reads showing significant similarity were subsequently mapped to the target locus using Geneious Prime v2025.1.3 and analyzed to identify nucleotide polymorphisms, which were used to reconstruct haplotypes of the AP2/ERF gene across cultivars.

### Reconstruction of AP2/ERF gene–encoded proteins in apricot

Protein sequences corresponding to the different allelic variants of the gene were obtained using the ExPASy Translate Tool (Duvaud et al. 2021). Structural visualization was performed through the AlphaFold Protein Structure Database (Jumper et al. 2021; Fleming et al. 2025) for the proteins associated with SSR108 and SSR114. For SSR111 and SSR120, three-dimensional protein models were generated using the AlphaFold2 Colab notebook.

### Data analysis

Color data are presented as mean ± standard deviation. Two approaches were used to calculate classification efficiency. The first method consisted of the proportion of correctly classified cultivars relative to the total number of cultivars. The second method applied a weighting factor of 0.5 to each cultivar category (yellow or orange) to account for the imbalance in sample size, as the number of orange cultivars exceeded that of yellow cultivars (Table S2).

## Supplementary Information

Below is the link to the electronic supplementary material.

Supplementary file1 (XLSX 11378 KB)

Supplementary file2 (XLSX 7059 KB)

Supplementary file3 (DOCX 124 KB)

Supplementary file4 (DOCX 19 KB)

Supplementary file5 (DOCX 120 KB)

## Data Availability

All genomic data used in this study are publicly available in the NCBI repository, and all datasets generated and analyzed during this work are presented within the article and its supplementary materials.
