# Supplementary material for: Genetic dissection of apricot fruit skin color (Prunus armeniaca L.) using SNP and SSR molecular markers
Source: Mol Breed. 2026 May 22;46(6):50. doi: 10.1007/s11032-026-01674-5 (PMC13197536; doi:10.1007/s11032-026-01674-5)
Supplement: Supplementary file 3 — Supplementary file3 (DOCX 124 KB) [file 11032_2026_1674_MOESM3_ESM.docx]

**Table S3.**

**Allelic sequences from genomes**

1. **“Currot” with SSR size of 108 bp**

TCAATTAGTTGACAAAAAGCAGATGGGTTGCAGATCAAGTGAGCTCCAAAGTGACAATTCACCATCACTACTCAGAGAAATATCCATTGAACTCAGTTGGGTTTCTTCCTCTTCTTTCTTCCCCATCACCACCTCCTGATATTCTTGTTTTGCAGCAGCAGCAGCTTCCTCTTTGTTCTTGTTACAGGCTCTCTGAAGGTTGCTTAAGGACTCAGATATGCCTCTCCCTTTGGCATTTCCTGATGATGATGAAGATCCAATCTTTAACTTTAAGCACTCAGGTATGCCATTGAAGGCATAGAGAGAAGCAGAGAACTTTCTTTCATATTTCATTCTCTTCATGGCTGCTCTTAGCATTGAAGGCTCATCTGCATAGGTCTCTGGCTCCACTGGCTCCTCTATCTCCTCAGCAATCTCAAAAGGAGATGGAGAGTAGTAGTTGGATGCTCCAATATCATCCACAAACTGGAAATCAATCACACCAACTTGATCATTGACTTCTTCTTCTTCTTGATCCATATCTTCCTCTGATCCTTCACCGACAAAATTTGCATCCCCGCCACTGTAAGTCTGTGCCACTTCACTCAAATTGCCATAGTCCATTTGATCAGTTTCTCTTGCAGTAGAGTACTCTTCCTTCTCAGTCAAGCAAGATTCAAAGCTTGATGTCATGTAGTCAATGCTACTTGCACTAATGATGTCATGATTGTTGCTTGTGATGTAATCTTCAGGATCATTAAGGAAATCAGTGTATTGGGTTTCTGAGAAATCTGCTCCTGCTTCTTCTTCCTGATGATCAGCATGTTGCTGCATTTGATGGTAGTTGATAGGCAAAGGAGCTGAAGGAGCAGAACAAGAGCTGCTGTTGTTTCTTGCTTTGAGCCTCTGCAGGAGAAGGTTTGTGATCTTTGAGGGAAGTGCTGGTGTTGAAGATGGTGAATGTGAGCAAGGCCAGAAATTGGTTCGAGTATTGGCACCGCGGAGCAAGCAAGCAGCCTCATCATAGGCCCTGGCTGCTTCCTCAGCAGTGTCAAATGTGCCCAACCACACTCTTATCTTTTGAATGGTGTCCTTAATCTCAGCCACCCATCTTCCGGAGGGTCTTTGGCGGACACCGACAAAGCGTTTTCGAGCTCTTCGTGCTCCTCCTAGCTCTGCAGCAGCCGAAGCTTCCTTCACCATCTCATCCCAAGCCATGGTTCCCTCACTTGAACTCCTGTCTTCCACTCCATCACTAACTTTTCTCTTCCTTGCCAT

1. **“Lito” with SSR size of 111 bp**

TCAATTAGTTGACAAAAAGCAGATGGGTTGCAGATCAAGTGAGCTCCAAAGTGACAATTCACCATCACTACTCAGAGAAATATCCATTGAACTCAGTTGGGTTTCTTCCTCTTCTTTCTTCCCCATCACCACCTCCTGATATTCTTGTTTTGCAGCAGCAGCAGCAGCTTCCTCTTTGTTCTTGTTACAGGCTCTCTGAAGGTTGCTTAAGGACTCAGATATGCCTCTCCCTTTGGCATTTCCTGATGATGATGAAGATCCAATCTTTAACTTTAAGCACTCAGGTATGCCATTGAAGGCATAGAGAGAAGCAGAGAACTTTCTTTCATATTTCATTCTCTTCATGGCTGCTCTTAGCATTGAAGGCTCATCTGCATAGGTCTCTGGCTCCACTGGCTCCTCTATCTCCTCAGCAATCTCAAAAGGAGATGGAGAGTAGTAGTTGGATGCTCCAATATCATCCACAAACTGGAAATCAATCACACCAACTTGATCATTGACTTCTTCTTCTTCTTGATCCATATCTTCCTCTGATCCTTCACCGACAAAATTTGCATCCCCGCCACTGTAAGTCTGTGCCACTTCACTCAAATTGCCATAGTCCATTTGATCAGTTTCTCTTGCAGTAGAGTACTCTTCCTTCTCAGTCAAGCAAGATTCAAAGCTTGATGTCATGTGGTCAATGCTACTTGCACTAATGATGTCATGATTGTTGCTTGTGATGTAATCTTCAGGATCATTAAGGAAATCAGTGTATTGGGTTTCTGAGAAATCTGCTCCTGCTTCTTCTTCCTGATGATCAGCATGTTGCTGCATTTGATGGTGGTTGATAGGCAAAGGAGCTGAAGGAGCAGAACAAGAGCTGCTGTTGTTTCTTGCTTTGAGCCTCTGCAGGAGAAGGTTTGTGATCTTTGAGGGAAGTGCTGGTGTTGAAGATGGTGAATGTGAGCAAGGCCAGAAATTGGTTCGAGTATTGGCACCGCGGAGCAAGCAAGCAGCCTCATCATAGGCCCTGGCTGCTTCCTCAGCAGTGTCAAATGTGCCCAACCACACTCTTATCTTTTGAATGGTGTCCTTAATCTCAGCCACCCATCTTCCGGAGGGTCTTTGGCGGACACCGACAAAGCGTTTTCGAGCTCTTCGTGCTCCTCCTAGCTCTGCAGCAGCCGAAGCTTCCTTCACCATCTCATCCCAAGCCATGGTTCCCTCACTTGAACTCCTGTCTTCCACTCCATCACTAACTTTTCTCTTCCTTGCCAT

1. **“Marouch n14” with SSR size of 108 bp**

TCAATTAGTTGACAAAAAGCAGATGGGTTGCAGATCAAGTGAGCTCCAAAGTGACAATTCACCATCACTACTCAGAGAAATATCCATTGAACTCAGTTGGGTTTCTTCCTCTTCTTTCTTCCCCATCACCACCTCCTGATATTCTTGTTTTGCAGCAGCAGCAGCTTCCTCTTTGTTCTTGTTACAGGCTCTCTGAAGGTTGCTTAAGGACTCAGATATGCCTCTCCCTTTGGCATTTCCTGATGATGATGAAGATCCAATCTTTAACTTTAAGCACTCAGGTATGCCATTGAAGGCATAGAGAGAAGCAGAGAACTTTCTTTCATATTTCATTCTCTTCATGGCTGCTCTTAGCATTGAAGGCTCATCTGCATAGGTCTCTGGCTCCACTGGCTCCTCTATCTCCTCAGCAATCTCAAAAGGAGATGGAGAGTAGTAGTTGGATGCTCCAATATCATCCACAAACTGGAAATCAATCACACCAACTTGATCATTGACTTCTTCTTCTTCTTGATCCATATCTTCCTCTGATCCTTCACCGACAAAATTTGCATCCCCGCCACTGTAAGTCTGTGCCACTTCACTCAAATTGCCATAGTCCATTTGATCAGTTTCTCTTGCAGTAGAGTACTCTTCCTTCTCAGTCAAGCAAGATTCAAAGCTTGATGTCATGTAGTCAATGCTACTTGCACTAATGATGTCATGATTGTTGCTTGTGATGTAATCTTCAGGATCATTAAGGAAATCAGTGTATTGGGTTTCTGAGAAATCTGCTCCTGCTTCTTCTTCCTGATGATCAGCATGTTGCTGCATTTGATGGTAGTTGATAGGCAAAGGAGCTGAAGGAGCAGAACAAGAGCTGCTGTTGTTTCTTGCTTTGAGCCTCTGCAGGAGAAGGTTTGTGATCTTTGAGGGAAGTGCTGGTGTTGAAGATGGTGAATGTGAGCAAGGCCAGAAATTGGTTCGAGTATTGGCACCGCGGAGCAAGCAAGCAGCCTCATCATAGGCCCTGGCTGCTTCCTCAGCAGTGTCAAATGTGCCCAACCACACTCTTATCTTTTGAATGGTGTCCTTAATCTCAGCCACCCATCTTCCGGAGGGTCTTTGGCGGACACCGACAAAGCGTTTTCGAGCTCTTCGTGCTCCTCCTAGCTCTGCAGCAGCCGAAGCTTCCTTCACCATCTCATCCCAAGCCATGGTTCCCTCACTTGAACTCCTGTCTTCCACTCCATCACTAACTTTTCTCTTCCTTGCCAT

1. **“Orange Red” with SSR size of 114 bp**

TCAATTAGTTGACAAAAAGCAGATGGGTTGCAGATCAAGTGAGCTCCAAAGTGACAATTCACCATCACTACTCAGAGAAATATCCATTGAACTCAGTTGGGTTTCTTCCTCTTCTTTCTTCCCCATCACCACCTCCTGATATTCTTGTTTTGCAGCAGCAGCAGCAGCAGCTTCCTCTTTGTTCTTGTTACAGGCTCTCTGAAGGTTGCTTAAGGACTCAGATATGCCTCTCCCTTTGGCATTTCCTGATGATGATGAAGATCCAATCTTTAACTTTAAGCACTCAGGTATGCCATTGAAGGCATAGAGAGAAGCAGAGAATTTTCTTTCATATTTCATTCTCTTCATGGCTGCTCTTAGCATTGAAGGCTCATCTGCATAGGTCTCTGGCTCCACTGGCTCCTCTATCTCCTCAGCAATCTCAAAAGGAGATGGAGAGTAGTAGTTGGATGCTCCAATATCATCCACAAACTGGAAATCAATCACACCAACTTGATCATTGACTTCTTCTTCTTCTTGATCCATATCTTCCTCTGATCCTTCACCGACAAAATTTGCATCCCCGCCACTGTAAGTCTGTGCCACTTCACTCAAATTTCCATAGTCCATTTGATCAGTTTCTCTTGCAGTAGAGTACTCTTCCTTCTCAGTCAAGCAAGATTCAAAGCTTGATGTCATGTAGTCAATGCTACTTGCACTAATGATGTCATGATTGTTGCTTGTGATGTAATCTTCAGGATCATTAAGGAAATCAGTGTATTGGGTTTCTGAGAAATCTGCTCCTGCTTCTTCTTCCTGATGATCAGCATGTTGCTGCATTTGATGGTGGTTGATAGGCAAAGGAGCTGAAGGAGCAGAACAAGAGCTGCTGTTGTTTCTTGCTTTGAGCCTCTGCAGGAGAAGGTTTGTGATCTTTGAGGGAAGTGCTGGTGTTGAAGATGGTGAATGTGAGCAAGGCCAGAAATTGGTTCGAGTATTGGCACCGCGGAGCAAGCAAGCAGCCTCATCATAGGCCCTGGCTGCTTCCTCAGCAGTGTCAAATGTGCCCAACCACACTCTTATCTTTTGAATGGTGTCCTTAATCTCAGCCACCCATCTTCCGGAGGGTCTTTGGCGGACACCGACAAAGCGTTTTCGAGCTCTTCGTGCTCCTCCTAGCTCTGCAGCAGCCGAAGCTTCCTTCACCATCTCATCCCAAGCCATGGTTCCCTCACTTGAACTCCTGTCTTCCACTCCATCACTAACTTTTCTCTTCCTTGCCAT

1. **“Stella” with SSR size of 108 bp**

TCAATTAGTTGACAAAAAGCAGATGGGTTGCAGATCAAGTGAGCTCCAAAGTGACAATTCACCATCACTACTCAGAGAAATATCCATTGAACTCAGTTGGGTTTCTTCCTCTTCTTTCTTCCCCATCACCACCTCCTGATATTCTTGTTTTGCAGCAGCAGCAGCTTCCTCTTTGTTCTTGTTACAGGCTCTCTGAAGGTTGCTTAAGGACTCAGATATGCCTCTCCCTTTGGCATTTCCTGATGATGATGAAGATCCAATCTTTAACTTTAAGCACTCAGGTATGCCATTGAAGGCATAGAGAGAAGCAGAGAACTTTCTTTCATATTTCATTCTCTTCATGGCTGCTCTTAGCATTGAAGGCTCATCTGCATAGGTCTCTGGCTCCACTGGCTCCTCTATCTCCTCAGCAATCTCAAAAGGAGATGGAGAGTAGTAGTTGGATGCTCCAATATCATCCACAAACTGGAAATCAATCACACCAACTTGATCATTGACTTCTTCTTCTTCTTGATCCATATCTTCCTCTGATCCTTCACCGACAAAATTTGCATCCCCGCCACTGTAAGTCTGTGCCACTTCACTCAAATTGCCATAGTCCATTTGATCAGTTTCTCTTGCAGTAGAGTACTCTTCCTTCTCAGTCAAGCAAGATTCAAAGCTTGATGTCATGTAGTCAATGCTACTTGCACTAATGATGTCATGATTGTTGCTTGTGATGTAATCTTCAGGATCATTAAGGAAATCAGTGTATTGGGTTTCTGAGAAATCTGCTCCTGCTTCTTCTTCCTGATGATCAGCATGTTGCTGCATTTGATGGTAGTTGATAGGCAAAGGAGCTGAAGGAGCAGAACAAGAGCTGCTGTTGTTTCTTGCTTTGAGCCTCTGCAGGAGAAGGTTTGTGATCTTTGAGGGAAGTGCTGGTGTTGAAGATGGTGAATGTGAGCAAGGCCAGAAATTGGTTCGAGTATTGGCACCGCGGAGCAAGCAAGCAGCCTCATCATAGGCCCTGGCTGCTTCCTCAGCAGTGTCAAATGTGCCCAACCACACTCTTATCTTTTGAATGGTGTCCTTAATCTCAGCCACCCATCTTCCGGAGGGTCTTTGGCGGACACCGACAAAGCGTTTTCGAGCTCTTCGTGCTCCTCCTAGCTCTGCAGCAGCCGAAGCTTCCTTCACCATCTCATCCCAAGCCATGGTTCCCTCACTTGAACTCCTGTCTTCCACTCCATCACTAACTTTTCTCTTCCTTGCCAT

**Reconstruction of allelic sequences from SRA data**

1. **“Bebeco” haplotype 1 and 2 with SSR size of 108 bp**

TCAATTAGTTGACAAAAAGCAGATGGGTTGCAGATCAAGTGAGCTCCAAAGTGACAATTCACCATCACTACTCAGAGAAATATCCATTGAACTCAGTTGGGTTTCTTCCTCTTCTTTCTTCCCCATCACCACCTCCTGATATTCTTGTTTTGCAGCAGCAGCAGCTTCCTCTTTGTTCTTGTTACAGGCTCTCTGAAGGTTGCTTAAGGACTCAGATATGCCTCTCCCTTTGGCATTTCCTGATGATGATGAAGATCCAATCTTTAACTTTAAGCACTCAGGTATGCCATTGAAGGCATAGAGAGAAGCAGAGAACTTTCTTTCATATTTCATTCTCTTCATGGCTGCTCTTAGCATTGAAGGCTCATCTGCATAGGTCTCTGGCTCCACTGGCTCCTCTATCTCCTCAGCAATCTCAAAAGGAGATGGAGAGTAGTAGTTGGATGCTCCAATATCATCCACAAACTGGAAATCAATCACACCAACTTGATCATTGACTTCTTCTTCTTCTTGATCCATATCTTCCTCTGATCCTTCACCGACAAAATTTGCATCCCCGCCACTGTAAGTCTGTGCCACTTCACTCAAATTGCCATAGTCCATTTGATCAGTTTCTCTTGCAGTAGAGTACTCTTCCTTCTCAGTCAAGCAAGATTCAAAGCTTGATGTCATGTAGTCAATGCTACTTGCACTAATGATGTCATGATTGTTGCTTGTGATGTAATCTTCAGGATCATTAAGGAAATCAGTGTATTGGGTTTCTGAGAAATCTGCTCCTGCTTCTTCTTCCTGATGATCAGCATGTTGCTGCATTTGATGGTAGTTGATAGGCAAAGGAGCTGAAGGAGCAGAACAAGAGCTGCTGTTGTTTCTTGCTTTGAGCCTCTGCAGGAGAAGGTTTGTGATCTTTGAGGGAAGTGCTGGTGTTGAAGATGGTGAATGTGAGCAAGGCCAGAAATTGGTTCGAGTATTGGCACCGCGGAGCAAGCAAGCAGCCTCATCATAGGCCCTGGCTGCTTCCTCAGCAGTGTCAAATGTGCCCAACCACACTCTTATCTTTTGAATGGTGTCCTTAATCTCAGCCACCCATCTTCCGGAGGGTCTTTGGCGGACACCGACAAAGCGTTTTCGAGCTCTTCGTGCTCCTCCTAGCTCTGCAGCAGCCGAAGCTTCCTTCACCATCTCATCCCAAGCCATGGTTCCCTCACTTGAACTCCTGTCTTCCACTCCATCACTAACTTTTCTCTTCCTTGCCAT

1. **“Bergeron” haplotype 1 with SSR size of 108 bp**

TCAATTAGTTGACAAAAAGCAGATGGGTTGCAGATCAAGTGAGCTCCAAAGTGACAATTCACCATCACTACTCAGAGAAATATCCATTGAACTCAGTTGGGTTTCTTCCTCTTCTTTCTTCCCCATCACCACCTCCTGATATTCTTGTTTTGCAGCAGCAGCAGCTTCCTCTTTGTTCTTGTTACAGGCTCTCTGAAGGTTGCTTAAGGACTCAGATATGCCTCTCCCTTTGGCATTTCCTGATGATGATGAAGATCCAATCTTTAACTTTAAGCACTCAGGTATGCCATTGAAGGCATAGAGAGAAGCAGAGAACTTTCTTTCATATTTCATTCTCTTCATGGCTGCTCTTAGCATTGAAGGCTCATCTGCATAGGTCTCTGGCTCCACTGGCTCCTCTATCTCCTCAGCAATCTCAAAAGGAGATGGAGAGTAGTAGTTGGATGCTCCAATATCATCCACAAACTGGAAATCAATCACACCAACTTGATCATTGACTTCTTCTTCTTCTTGATCCATATCTTCCTCTGATCCTTCACCGACAAAATTTGCATCCCCGCCACTGTAAGTCTGTGCCACTTCACTCAAATTGCCATAGTCCATTTGATCAGTTTCTCTTGCAGTAGAGTACTCTTCCTTCTCAGTCAAGCAAGATTCAAAGCTTGATGTCATGTAGTCAATGCTACTTGCACTAATGATGTCATGATTGTTGCTTGTGATGTAATCTTCAGGATCATTAAGGAAATCAGTGTATTGGGTTTCTGAGAAATCTGCTCCTGCTTCTTCTTCCTGATGATCAGCATGTTGCTGCATTTGATGGTAGTTGATAGGCAAAGGAGCTGAAGGAGCAGAACAAGAGCTGCTGTTGTTTCTTGCTTTGAGCCTCTGCAGGAGAAGGTTTGTGATCTTTGAGGGAAGTGCTGGTGTTGAAGATGGTGAATGTGAGCAAGGCCAGAAATTGGTTCGAGTATTGGCACCGCGGAGCAAGCAAGCAGCCTCATCATAGGCCCTGGCTGCTTCCTCAGCAGTGTCAAATGTGCCCAACCACACTCTTATCTTTTGAATGGTGTCCTTAATCTCAGCCACCCATCTTCCGGAGGGTCTTTGGCGGACACCGACAAAGCGTTTTCGAGCTCTTCGTGCTCCTCCTAGCTCTGCAGCAGCCGAAGCTTCCTTCACCATCTCATCCCAAGCCATGGTTCCCTCACTTGAACTCCTGTCTTCCACTCCATCACTAACTTTTCTCTTCCTTGCCAT

1. **“Bergeron” haplotype 2 with SSR size of 120 bp**

TCAATTAGTTGACAAAAAGCAGATGGGTTGCAGATCAAGTGAGCTCCAAAGTGACAATTCACCATCACTACTCAGAGAAATATCCATTGAACTCAGTTGGGTTTCTTCCTCTTCTTTCTTCCCCATCACCACCTCCTGATATTCTTGTTTTGCAGCAGCAGCAGCAGCAGCAGCAGCTTCCTCTTTGTTCTTGTTACAGGCTCTCTGAAGGTTGCTTAAGGACTCAGATATGCCTCTCCCTTTGGCATTTCCTGATGATGATGAAGATCCAATCTTTAACTTTAAGCACTCAGGTATGCCATTGAAGGCATAGAGAGAAGCAGAGAACTTTCTTTCATATTTCATTCTCTTCATGGCTGCTCTTAGCATTGAAGGCTCATCTGCATAGGTCTCTGGCTCCACTGGCTCCTCTATCTCCTCAGCAATCTCAAAAGGAGATGGAGAGTAGTAGTTGGATGCTCCAATATCATCCACAAACTGGAAATCAATCACACCAACTTGATCATTGACTTCTTCTTCTTCTTGATCCATATCTTCCTCTGATCCTTCACCGACAAAATTTGCATCCCCGCCACTGTAAGTCTGTGCCACTTCACTCAAATTGCCATAGTCCATTTGATCAGTTTCTCTTGCAGTAGAGTACTCTTCCTTCTCAGTCAAGCAAGATTCAAAGCTTGATGTCATGTAGTCAATGCTACTTGCACTAATGATGTCATGATTGTTGCTTGTGATGTAATCTTCAGGATCATTAAGGAAATCAGTGTATTGGGTTTCTGAGAAATCTGCTCCTGCTTCTTCTTCCTGATGATCAGCATGTTGCTGCATTTGATGGTGGTTGATAGGCAAAGGAGCTGAAGGAGCAGAACAAGAGCTGCTGTTGTTTCTTGCTTTGAGCCTCTGCAGGAGAAGGTTTGTGATCTTTGAGGGAAGTGCTGGTGTTGAAGATGGTGAATGTGAGCAAGGCCAGAAATTGGTTCGAGTATTGGCACCGCGGAGCAAGCAAGCAGCCTCATCATAGGCCCTGGCTGCTTCCTCAGCAGTGTCAAATGTGCCCAACCACACTCTTATCTTTTGAATGGTGTCCTTAATCTCAGCCACCCATCTTCCGGAGGGTCTTTGGCGGACACCGACAAAGCGTTTTCGAGCTCTTCGTGCTCCTCCTAGCTCTGCAGCAGCCGAAGCTTCCTTCACCATCTCATCCCAAGCCATGGTTCCCTCACTTGAACTCCTGTCTTCCACTCCATCACTAACTTTTCTCTTCCTTGCCAT

1. **“Canino” haplotype 1 and 2 with SSR size of 108 bp**

TCAATTAGTTGACAAAAAGCAGATGGGTTGCAGATCAAGTGAGCTCCAAAGTGACAATTCACCATCACTACTCAGAGAAATATCCATTGAACTCAGTTGGGTTTCTTCCTCTTCTTTCTTCCCCATCACCACCTCCTGATATTCTTGTTTTGCAGCAGCAGCAGCTTCCTCTTTGTTCTTGTTACAGGCTCTCTGAAGGTTGCTTAAGGACTCAGATATGCCTCTCCCTTTGGCATTTCCTGATGATGATGAAGATCCAATCTTTAACTTTAAGCACTCAGGTATGCCATTGAAGGCATAGAGAGAAGCAGAGAACTTTCTTTCATATTTCATTCTCTTCATGGCTGCTCTTAGCATTGAAGGCTCATCTGCATAGGTCTCTGGCTCCACTGGCTCCTCTATCTCCTCAGCAATCTCAAAAGGAGATGGAGAGTAGTAGTTGGATGCTCCAATATCATCCACAAACTGGAAATCAATCACACCAACTTGATCATTGACTTCTTCTTCTTCTTGATCCATATCTTCCTCTGATCCTTCACCGACAAAATTTGCATCCCCGCCACTGTAAGTCTGTGCCACTTCACTCAAATTGCCATAGTCCATTTGATCAGTTTCTCTTGCAGTAGAGTACTCTTCCTTCTCAGTCAAGCAAGATTCAAAGCTTGATGTCATGTAGTCAATGCTACTTGCACTAATGATGTCATGATTGTTGCTTGTGATGTAATCTTCAGGATCATTAAGGAAATCAGTGTATTGGGTTTCTGAGAAATCTGCTCCTGCTTCTTCTTCCTGATGATCAGCATGTTGCTGCATTTGATGGTAGTTGATAGGCAAAGGAGCTGAAGGAGCAGAACAAGAGCTGCTGTTGTTTCTTGCTTTGAGCCTCTGCAGGAGAAGGTTTGTGATCTTTGAGGGAAGTGCTGGTGTTGAAGATGGTGAATGTGAGCAAGGCCAGAAATTGGTTCGAGTATTGGCACCGCGGAGCAAGCAAGCAGCCTCATCATAGGCCCTGGCTGCTTCCTCAGCAGTGTCAAATGTGCCCAACCACACTCTTATCTTTTGAATGGTGTCCTTAATCTCAGCCACCCATCTTCCGGAGGGTCTTTGGCGGACACCGACAAAGCGTTTTCGAGCTCTTCGTGCTCCTCCTAGCTCTGCAGCAGCCGAAGCTTCCTTCACCATCTCATCCCAAGCCATGGTTCCCTCACTTGAACTCCTGTCTTCCACTCCATCACTAACTTTTCTCTTCCTTGCCAT

1. **“Moniqui” haplotype 1 and 2 with SSR size of 108 bp**

TCAATTAGTTGACAAAAAGCAGATGGGTTGCAGATCAAGTGAGCTCCAAAGTGACAATTCACCATCACTACTCAGAGAAATATCCATTGAACTCAGTTGGGTTTCTTCCTCTTCTTTCTTCCCCATCACCACCTCCTGATATTCTTGTTTTGCAGCAGCAGCAGCTTCCTCTTTGTTCTTGTTACAGGCTCTCTGAAGGTTGCTTAAGGACTCAGATATGCCTCTCCCTTTGGCATTTCCTGATGATGATGAAGATCCAATCTTTAACTTTAAGCACTCAGGTATGCCATTGAAGGCATAGAGAGAAGCAGAGAACTTTCTTTCATATTTCATTCTCTTCATGGCTGCTCTTAGCATTGAAGGCTCATCTGCATAGGTCTCTGGCTCCACTGGCTCCTCTATCTCCTCAGCAATCTCAAAAGGAGATGGAGAGTAGTAGTTGGATGCTCCAATATCATCCACAAACTGGAAATCAATCACACCAACTTGATCATTGACTTCTTCTTCTTCTTGATCCATATCTTCCTCTGATCCTTCACCGACAAAATTTGCATCCCCGCCACTGTAAGTCTGTGCCACTTCACTCAAATTGCCATAGTCCATTTGATCAGTTTCTCTTGCAGTAGAGTACTCTTCCTTCTCAGTCAAGCAAGATTCAAAGCTTGATGTCATGTAGTCAATGCTACTTGCACTAATGATGTCATGATTGTTGCTTGTGATGTAATCTTCAGGATCATTAAGGAAATCAGTGTATTGGGTTTCTGAGAAATCTGCTCCTGCTTCTTCTTCCTGATGATCAGCATGTTGCTGCATTTGATGGTAGTTGATAGGCAAAGGAGCTGAAGGAGCAGAACAAGAGCTGCTGTTGTTTCTTGCTTTGAGCCTCTGCAGGAGAAGGTTTGTGATCTTTGAGGGAAGTGCTGGTGTTGAAGATGGTGAATGTGAGCAAGGCCAGAAATTGGTTCGAGTATTGGCACCGCGGAGCAAGCAAGCAGCCTCATCATAGGCCCTGGCTGCTTCCTCAGCAGTGTCAAATGTGCCCAACCACACTCTTATCTTTTGAATGGTGTCCTTAATCTCAGCCACCCATCTTCCGGAGGGTCTTTGGCGGACACCGACAAAGCGTTTTCGAGCTCTTCGTGCTCCTCCTAGCTCTGCAGCAGCCGAAGCTTCCTTCACCATCTCATCCCAAGCCATGGTTCCCTCACTTGAACTCCTGTCTTCCACTCCATCACTAACTTTTCTCTTCCTTGCCAT

1. **“Murciana” haplotype 1 with SSR size of 108 bp**

TCAATTAGTTGACAAAAAGCAGATGGGTTGCAGATCAAGTGAGCTCCAAAGTGACAATTCACCATCACTACTCAGAGAAATATCCATTGAACTCAGTTGGGTTTCTTCCTCTTCTTTCTTCCCCATCACCACCTCCTGATATTCTTGTTTTGCAGCAGCAGCAGCTTCCTCTTTGTTCTTGTTACAGGCTCTCTGAAGGTTGCTTAAGGACTCAGATATGCCTCTCCCTTTGGCATTTCCTGATGATGATGAAGATCCAATCTTTAACTTTAAGCACTCAGGTATGCCATTGAAGGCATAGAGAGAAGCAGAGAACTTTCTTTCATATTTCATTCTCTTCATGGCTGCTCTTAGCATTGAAGGCTCATCTGCATAGGTCTCTGGCTCCACTGGCTCCTCTATCTCCTCAGCAATCTCAAAAGGAGATGGAGAGTAGTAGTTGGATGCTCCAATATCATCCACAAACTGGAAATCAATCACACCAACTTGATCATTGACTTCTTCTTCTTCTTGATCCATATCTTCCTCTGATCCTTCACCGACAAAATTTGCATCCCCGCCACTGTAAGTCTGTGCCACTTCACTCAAATTGCCATAGTCCATTTGATCAGTTTCTCTTGCAGTAGAGTACTCTTCCTTCTCAGTCAAGCAAGATTCAAAGCTTGATGTCATGTAGTCAATGCTACTTGCACTAATGATGTCATGATTGTTGCTTGTGATGTAATCTTCAGGATCATTAAGGAAATCAGTGTATTGGGTTTCTGAGAAATCTGCTCCTGCTTCTTCTTCCTGATGATCAGCATGTTGCTGCATTTGATGGTAGTTGATAGGCAAAGGAGCTGAAGGAGCAGAACAAGAGCTGCTGTTGTTTCTTGCTTTGAGCCTCTGCAGGAGAAGGTTTGTGATCTTTGAGGGAAGTGCTGGTGTTGAAGATGGTGAATGTGAGCAAGGCCAGAAATTGGTTCGAGTATTGGCACCGCGGAGCAAGCAAGCAGCCTCATCATAGGCCCTGGCTGCTTCCTCAGCAGTGTCAAATGTGCCCAACCACACTCTTATCTTTTGAATGGTGTCCTTAATCTCAGCCACCCATCTTCCGGAGGGTCTTTGGCGGACACCGACAAAGCGTTTTCGAGCTCTTCGTGCTCCTCCTAGCTCTGCAGCAGCCGAAGCTTCCTTCACCATCTCATCCCAAGCCATGGTTCCCTCACTTGAACTCCTGTCTTCCACTCCATCACTAACTTTTCTCTTCCTTGCCAT

1. **“Murciana” haplotype 2 with SSR size of 114 bp**

TCAATTAGTTGACAAAAAGCAGATGGGTTGCAGATCAAGTGAGCTCCAAAGTGACAATTCACCATCACTACTCAGAGAAATATCCATTGAACTCAGTTGGGTTTCTTCCTCTTCTTTCTTCCCCATCACCACCTCCTGATATTCTTGTTTTGCAGCAGCAGCAGCAGCAGCTTCCTCTTTGTTCTTGTTACAGGCTCTCTGAAGGTTGCTTAAGGACTCAGATATGCCTCTCCCTTTGGCATTTCCTGATGATGATGAAGATCCAATCTTTAACTTTAAGCACTCAGGTATGCCATTGAAGGCATAGAGAGAAGCAGAGAATTTTCTTTCATATTTCATTCTCTTCATGGCTGCTCTTAGCATTGAAGGCTCATCTGCATAGGTCTCTGGCTCCACTGGCTCCTCTATCTCCTCAGCAATCTCAAAAGGAGATGGAGAGTAGTAGTTGGATGCTCCAATATCATCCACAAACTGGAAATCAATCACACCAACTTGATCATTGACTTCTTCTTCTTCTTGATCCATATCTTCCTCTGATCCTTCACCGACAAAATTTGCATCCCCGCCACTGTAAGTCTGTGCCACTTCACTCAAATTTCCATAGTCCATTTGATCAGTTTCTCTTGCAGTAGAGTACTCTTCCTTCTCAGTCAAGCAAGATTCAAAGCTTGATGTCATGTAGTCAATGCTACTTGCACTAATGATGTCATGATTGTTGCTTGTGATGTAATCTTCAGGATCATTAAGGAAATCAGTGTATTGGGTTTCTGAGAAATCTGCTCCTGCTTCTTCTTCCTGATGATCAGCATGTTGCTGCATTTGATGGTGGTTGATAGGCAAAGGAGCTGAAGGAGCAGAACAAGAGCTGCTGTTGTTTCTTGCTTTGAGCCTCTGCAGGAGAAGGTTTGTGATCTTTGAGGGAAGTGCTGGTGTTGAAGATGGTGAATGTGAGCAAGGCCAGAAATTGGTTCGAGTATTGGCACCGCGGAGCAAGCAAGCAGCCTCATCATAGGCCCTGGCTGCTTCCTCAGCAGTGTCAAATGTGCCCAACCACACTCTTATCTTTTGAATGGTGTCCTTAATCTCAGCCACCCATCTTCCGGAGGGTCTTTGGCGGACACCGACAAAGCGTTTTCGAGCTCTTCGTGCTCCTCCTAGCTCTGCAGCAGCCGAAGCTTCCTTCACCATCTCATCCCAAGCCATGGTTCCCTCACTTGAACTCCTGTCTTCCACTCCATCACTAACTTTTCTCTTCCTTGCCAT

1. **“Orange Red” haplotype 1 with SSR size of 114 bp**

TCAATTAGTTGACAAAAAGCAGATGGGTTGCAGATCAAGTGAGCTCCAAAGTGACAATTCACCATCACTACTCAGAGAAATATCCATTGAACTCAGTTGGGTTTCTTCCTCTTCTTTCTTCCCCATCACCACCTCCTGATATTCTTGTTTTGCAGCAGCAGCAGCAGCAGCTTCCTCTTTGTTCTTGTTACAGGCTCTCTGAAGGTTGCTTAAGGACTCAGATATGCCTCTCCCTTTGGCATTTCCTGATGATGATGAAGATCCAATCTTTAACTTTAAGCACTCAGGTATGCCATTGAAGGCATAGAGAGAAGCAGAGAATTTTCTTTCATATTTCATTCTCTTCATGGCTGCTCTTAGCATTGAAGGCTCATCTGCATAGGTCTCTGGCTCCACTGGCTCCTCTATCTCCTCAGCAATCTCAAAAGGAGATGGAGAGTAGTAGTTGGATGCTCCAATATCATCCACAAACTGGAAATCAATCACACCAACTTGATCATTGACTTCTTCTTCTTCTTGATCCATATCTTCCTCTGATCCTTCACCGACAAAATTTGCATCCCCGCCACTGTAAGTCTGTGCCACTTCACTCAAATTTCCATAGTCCATTTGATCAGTTTCTCTTGCAGTAGAGTACTCTTCCTTCTCAGTCAAGCAAGATTCAAAGCTTGATGTCATGTAGTCAATGCTACTTGCACTAATGATGTCATGATTGTTGCTTGTGATGTAATCTTCAGGATCATTAAGGAAATCAGTGTATTGGGTTTCTGAGAAATCTGCTCCTGCTTCTTCTTCCTGATGATCAGCATGTTGCTGCATTTGATGGTGGTTGATAGGCAAAGGAGCTGAAGGAGCAGAACAAGAGCTGCTGTTGTTTCTTGCTTTGAGCCTCTGCAGGAGAAGGTTTGTGATCTTTGAGGGAAGTGCTGGTGTTGAAGATGGTGAATGTGAGCAAGGCCAGAAATTGGTTCGAGTATTGGCACCGCGGAGCAAGCAAGCAGCCTCATCATAGGCCCTGGCTGCTTCCTCAGCAGTGTCAAATGTGCCCAACCACACTCTTATCTTTTGAATGGTGTCCTTAATCTCAGCCACCCATCTTCCGGAGGGTCTTTGGCGGACACCGACAAAGCGTTTTCGAGCTCTTCGTGCTCCTCCTAGCTCTGCAGCAGCCGAAGCTTCCTTCACCATCTCATCCCAAGCCATGGTTCCCTCACTTGAACTCCTGTCTTCCACTCCATCACTAACTTTTCTCTTCCTTGCCAT

1. **“Orange Red” haplotype 2 with SSR size of 120 bp**

TCAATTAGTTGACAAAAAGCAGATGGGTTGCAGATCAAGTGAGCTCCAAAGTGACAATTCACCATCACTACTCAGAGAAATATCCATTGAACTCAGTTGGGTTTCTTCCTCTTCTTTCTTCCCCATCACCACCTCCTGATATTCTTGTTTTGCAGCAGCAGCAGCAGCAGCAGCAGCTTCCTCTTTGTTCTTGTTACAGGCTCTCTGAAGGTTGCTTAAGGACTCAGATATGCCTCTCCCTTTGGCATTTCCTGATGATGATGAAGATCCAATCTTTAACTTTAAGCACTCAGGTATGCCATTGAAGGCATAGAGAGAAGCAGAGAACTTTCTTTCATATTTCATTCTCTTCATGGCTGCTCTTAGCATTGAAGGCTCATCTGCATAGGTCTCTGGCTCCACTGGCTCCTCTATCTCCTCAGCAATCTCAAAAGGAGATGGAGAGTAGTAGTTGGATGCTCCAATATCATCCACAAACTGGAAATCAATCACACCAACTTGATCATTGACTTCTTCTTCTTCTTGATCCATATCTTCCTCTGATCCTTCACCGACAAAATTTGCATCCCCGCCACTGTAAGTCTGTGCCACTTCACTCAAATTGCCATAGTCCATTTGATCAGTTTCTCTTGCAGTAGAGTACTCTTCCTTCTCAGTCAAGCAAGATTCAAAGCTTGATGTCATGTAGTCAATGCTACTTGCACTAATGATGTCATGATTGTTGCTTGTGATGTAATCTTCAGGATCATTAAGGAAATCAGTGTATTGGGTTTCTGAGAAATCTGCTCCTGCTTCTTCTTCCTGATGATCAGCATGTTGCTGCATTTGATGGTGGTTGATAGGCAAAGGAGCTGAAGGAGCAGAACAAGAGCTGCTGTTGTTTCTTGCTTTGAGCCTCTGCAGGAGAAGGTTTGTGATCTTTGAGGGAAGTGCTGGTGTTGAAGATGGTGAATGTGAGCAAGGCCAGAAATTGGTTCGAGTATTGGCACCGCGGAGCAAGCAAGCAGCCTCATCATAGGCCCTGGCTGCTTCCTCAGCAGTGTCAAATGTGCCCAACCACACTCTTATCTTTTGAATGGTGTCCTTAATCTCAGCCACCCATCTTCCGGAGGGTCTTTGGCGGACACCGACAAAGCGTTTTCGAGCTCTTCGTGCTCCTCCTAGCTCTGCAGCAGCCGAAGCTTCCTTCACCATCTCATCCCAAGCCATGGTTCCCTCACTTGAACTCCTGTCTTCCACTCCATCACTAACTTTTCTCTTCCTTGCCAT

1. **“Palsteyn” haplotype 1 with SSR size of 108 bp**

TCAATTAGTTGACAAAAAGCAGATGGGTTGCAGATCAAGTGAGCTCCAAAGTGACAATTCACCATCACTACTCAGAGAAATATCCATTGAACTCAGTTGGGTTTCTTCCTCTTCTTTCTTCCCCATCACCACCTCCTGATATTCTTGTTTTGCAGCAGCAGCAGCTTCCTCTTTGTTCTTGTTACAGGCTCTCTGAAGGTTGCTTAAGGACTCAGATATGCCTCTCCCTTTGGCATTTCCTGATGATGATGAAGATCCAATCTTTAACTTTAAGCACTCAGGTATGCCATTGAAGGCATAGAGAGAAGCAGAGAACTTTCTTTCATATTTCATTCTCTTCATGGCTGCTCTTAGCATTGAAGGCTCATCTGCATAGGTCTCTGGCTCCACTGGCTCCTCTATCTCCTCAGCAATCTCAAAAGGAGATGGAGAGTAGTAGTTGGATGCTCCAATATCATCCACAAACTGGAAATCAATCACACCAACTTGATCATTGACTTCTTCTTCTTCTTGATCCATATCTTCCTCTGATCCTTCACCGACAAAATTTGCATCCCCGCCACTGTAAGTCTGTGCCACTTCACTCAAATTGCCATAGTCCATTTGATCAGTTTCTCTTGCAGTAGAGTACTCTTCCTTCTCAGTCAAGCAAGATTCAAAGCTTGATGTCATGTAGTCAATGCTACTTGCACTAATGATGTCATGATTGTTGCTTGTGATGTAATCTTCAGGATCATTAAGGAAATCAGTGTATTGGGTTTCTGAGAAATCTGCTCCTGCTTCTTCTTCCTGATGATCAGCATGTTGCTGCATTTGATGGTAGTTGATAGGCAAAGGAGCTGAAGGAGCAGAACAAGAGCTGCTGTTGTTTCTTGCTTTGAGCCTCTGCAGGAGAAGGTTTGTGATCTTTGAGGGAAGTGCTGGTGTTGAAGATGGTGAATGTGAGCAAGGCCAGAAATTGGTTCGAGTATTGGCACCGCGGAGCAAGCAAGCAGCCTCATCATAGGCCCTGGCTGCTTCCTCAGCAGTGTCAAATGTGCCCAACCACACTCTTATCTTTTGAATGGTGTCCTTAATCTCAGCCACCCATCTTCCGGAGGGTCTTTGGCGGACACCGACAAAGCGTTTTCGAGCTCTTCGTGCTCCTCCTAGCTCTGCAGCAGCCGAAGCTTCCTTCACCATCTCATCCCAAGCCATGGTTCCCTCACTTGAACTCCTGTCTTCCACTCCATCACTAACTTTTCTCTTCCTTGCCAT

1. **“Palsteyn” haplotype 2 with SSR size of 111 bp**

TCAATTAGTTGACAAAAAGCAGATGGGTTGCAGATCAAGTGAGCTCCAAAGTGACAATTCACCATCACTACTCAGAGAAATATCCATTGAACTCAGTTGGGTTTCTTCCTCTTCTTTCTTCCCCATCACCACCTCCTGATATTCTTGTTTTGCAGCAGCAGCAGCAGCTTCCTCTTTGTTCTTGTTACAGGCTCTCTGAAGGTTGCTTAAGGACTCAGATATGCCTCTCCCTTTGGCATTTCCTGATGATGATGAAGATCCAATCTTTAACTTTAAGCACTCAGGTATGCCATTGAAGGCATAGAGAGAAGCAGAGAACTTTCTTTCATATTTCATTCTCTTCATGGCTGCTCTTAGCATTGAAGGCTCATCTGCATAGGTCTCTGGCTCCACTGGCTCCTCTATCTCCTCAGCAATCTCAAAAGGAGATGGAGAGTAGTAGTTGGATGCTCCAATATCATCCACAAACTGGAAATCAATCACACCAACTTGATCATTGACTTCTTCTTCTTCTTGATCCATATCTTCCTCTGATCCTTCACCGACAAAATTTGCATCCCCGCCACTGTAAGTCTGTGCCACTTCACTCAAATTGCCATAGTCCATTTGATCAGTTTCTCTTGCAGTAGAGTACTCTTCCTTCTCAGTCAAGCAAGATTCAAAGCTTGATGTCATGTGGTCAATGCTACTTGCACTAATGATGTCATGATTGTTGCTTGTGATGTAATCTTCAGGATCATTAAGGAAATCAGTGTATTGGGTTTCTGAGAAATCTGCTCCTGCTTCTTCTTCCTGATGATCAGCATGTTGCTGCATTTGATGGTGGTTGATAGGCAAAGGAGCTGAAGGAGCAGAACAAGAGCTGCTGTTGTTTCTTGCTTTGAGCCTCTGCAGGAGAAGGTTTGTGATCTTTGAGGGAAGTGCTGGTGTTGAAGATGGTGAATGTGAGCAAGGCCAGAAATTGGTTCGAGTATTGGCACCGCGGAGCAAGCAAGCAGCCTCATCATAGGCCCTGGCTGCTTCCTCAGCAGTGTCAAATGTGCCCAACCACACTCTTATCTTTTGAATGGTGTCCTTAATCTCAGCCACCCATCTTCCGGAGGGTCTTTGGCGGACACCGACAAAGCGTTTTCGAGCTCTTCGTGCTCCTCCTAGCTCTGCAGCAGCCGAAGCTTCCTTCACCATCTCATCCCAAGCCATGGTTCCCTCACTTGAACTCCTGTCTTCCACTCCATCACTAACTTTTCTCTTCCTTGCCAT

1. **“San Castrese” haplotype 1 with SSR size of 108 bp**

TCAATTAGTTGACAAAAAGCAGATGGGTTGCAGATCAAGTGAGCTCCAAAGTGACAATTCACCATCACTACTCAGAGAAATATCCATTGAACTCAGTTGGGTTTCTTCCTCTTCTTTCTTCCCCATCACCACCTCCTGATATTCTTGTTTTGCAGCAGCAGCAGCTTCCTCTTTGTTCTTGTTACAGGCTCTCTGAAGGTTGCTTAAGGACTCAGATATGCCTCTCCCTTTGGCATTTCCTGATGATGATGAAGATCCAATCTTTAACTTTAAGCACTCAGGTATGCCATTGAAGGCATAGAGAGAAGCAGAGAACTTTCTTTCATATTTCATTCTCTTCATGGCTGCTCTTAGCATTGAAGGCTCATCTGCATAGGTCTCTGGCTCCACTGGCTCCTCTATCTCCTCAGCAATCTCAAAAGGAGATGGAGAGTAGTAGTTGGATGCTCCAATATCATCCACAAACTGGAAATCAATCACACCAACTTGATCATTGACTTCTTCTTCTTCTTGATCCATATCTTCCTCTGATCCTTCACCGACAAAATTTGCATCCCCGCCACTGTAAGTCTGTGCCACTTCACTCAAATTGCCATAGTCCATTTGATCAGTTTCTCTTGCAGTAGAGTACTCTTCCTTCTCAGTCAAGCAAGATTCAAAGCTTGATGTCATGTAGTCAATGCTACTTGCACTAATGATGTCATGATTGTTGCTTGTGATGTAATCTTCAGGATCATTAAGGAAATCAGTGTATTGGGTTTCTGAGAAATCTGCTCCTGCTTCTTCTTCCTGATGATCAGCATGTTGCTGCATTTGATGGTAGTTGATAGGCAAAGGAGCTGAAGGAGCAGAACAAGAGCTGCTGTTGTTTCTTGCTTTGAGCCTCTGCAGGAGAAGGTTTGTGATCTTTGAGGGAAGTGCTGGTGTTGAAGATGGTGAATGTGAGCAAGGCCAGAAATTGGTTCGAGTATTGGCACCGCGGAGCAAGCAAGCAGCCTCATCATAGGCCCTGGCTGCTTCCTCAGCAGTGTCAAATGTGCCCAACCACACTCTTATCTTTTGAATGGTGTCCTTAATCTCAGCCACCCATCTTCCGGAGGGTCTTTGGCGGACACCGACAAAGCGTTTTCGAGCTCTTCGTGCTCCTCCTAGCTCTGCAGCAGCCGAAGCTTCCTTCACCATCTCATCCCAAGCCATGGTTCCCTCACTTGAACTCCTGTCTTCCACTCCATCACTAACTTTTCTCTTCCTTGCCAT

1. **“San Castrese” haplotype 2 with SSR size of 111 bp**

TCAATTAGTTGACAAAAAGCAGATGGGTTGCAGATCAAGTGAGCTCCAAAGTGACAATTCACCATCACTACTCAGAGAAATATCCATTGAACTCAGTTGGGTTTCTTCCTCTTCTTTCTTCCCCATCACCACCTCCTGATATTCTTGTTTTGCAGCAGCAGCAGCAGCTTCCTCTTTGTTCTTGTTACAGGCTCTCTGAAGGTTGCTTAAGGACTCAGATATGCCTCTCCCTTTGGCATTTCCTGATGATGATGAAGATCCAATCTTTAACTTTAAGCACTCAGGTATGCCATTGAAGGCATAGAGAGAAGCAGAGAACTTTCTTTCATATTTCATTCTCTTCATGGCTGCTCTTAGCATTGAAGGCTCATCTGCATAGGTCTCTGGCTCCACTGGCTCCTCTATCTCCTCAGCAATCTCAAAAGGAGATGGAGAGTAGTAGTTGGATGCTCCAATATCATCCACAAACTGGAAATCAATCACACCAACTTGATCATTGACTTCTTCTTCTTCTTGATCCATATCTTCCTCTGATCCTTCACCGACAAAATTTGCATCCCCGCCACTGTAAGTCTGTGCCACTTCACTCAAATTGCCATAGTCCATTTGATCAGTTTCTCTTGCAGTAGAGTACTCTTCCTTCTCAGTCAAGCAAGATTCAAAGCTTGATGTCATGTGGTCAATGCTACTTGCACTAATGATGTCATGATTGTTGCTTGTGATGTAATCTTCAGGATCATTAAGGAAATCAGTGTATTGGGTTTCTGAGAAATCTGCTCCTGCTTCTTCTTCCTGATGATCAGCATGTTGCTGCATTTGATGGTGGTTGATAGGCAAAGGAGCTGAAGGAGCAGAACAAGAGCTGCTGTTGTTTCTTGCTTTGAGCCTCTGCAGGAGAAGGTTTGTGATCTTTGAGGGAAGTGCTGGTGTTGAAGATGGTGAATGTGAGCAAGGCCAGAAATTGGTTCGAGTATTGGCACCGCGGAGCAAGCAAGCAGCCTCATCATAGGCCCTGGCTGCTTCCTCAGCAGTGTCAAATGTGCCCAACCACACTCTTATCTTTTGAATGGTGTCCTTAATCTCAGCCACCCATCTTCCGGAGGGTCTTTGGCGGACACCGACAAAGCGTTTTCGAGCTCTTCGTGCTCCTCCTAGCTCTGCAGCAGCCGAAGCTTCCTTCACCATCTCATCCCAAGCCATGGTTCCCTCACTTGAACTCCTGTCTTCCACTCCATCACTAACTTTTCTCTTCCTTGCCAT
