# Supplementary material for: Genetic dissection of apricot fruit skin color (Prunus armeniaca L.) using SNP and SSR molecular markers
Source: Mol Breed. 2026 May 22;46(6):50. doi: 10.1007/s11032-026-01674-5 (PMC13197536; doi:10.1007/s11032-026-01674-5)
Supplement: Supplementary file 4 — Supplementary file4 (DOCX 19 KB) [file 11032_2026_1674_MOESM4_ESM.docx]

**Table S4.**

CLUSTAL 2.1 multiple sequence alignment

SSR108 TCAATTAGTTGACAAAAAGCAGATGGGTTGCAGATCAAGTGAGCTCCAAAGTGACAATTC

SSR111 TCAATTAGTTGACAAAAAGCAGATGGGTTGCAGATCAAGTGAGCTCCAAAGTGACAATTC

SSR114 TCAATTAGTTGACAAAAAGCAGATGGGTTGCAGATCAAGTGAGCTCCAAAGTGACAATTC

SSR120 TCAATTAGTTGACAAAAAGCAGATGGGTTGCAGATCAAGTGAGCTCCAAAGTGACAATTC

************************************************************

SSR108 ACCATCACTACTCAGAGAAATATCCATTGAACTCAGTTGGGTTTCTTCCTCTTCTTTCTT

SSR111 ACCATCACTACTCAGAGAAATATCCATTGAACTCAGTTGGGTTTCTTCCTCTTCTTTCTT

SSR114 ACCATCACTACTCAGAGAAATATCCATTGAACTCAGTTGGGTTTCTTCCTCTTCTTTCTT

SSR120 ACCATCACTACTCAGAGAAATATCCATTGAACTCAGTTGGGTTTCTTCCTCTTCTTTCTT

************************************************************

SSR108 CCCCATCACCACCTCCTGATATTCTTGTTTTGCAGCAGCAGCAGC------------TTC

SSR111 CCCCATCACCACCTCCTGATATTCTTGTTTTGCAGCAGCAGCAGCAGC---------TTC

SSR114 CCCCATCACCACCTCCTGATATTCTTGTTTTGCAGCAGCAGCAGCAGCAGC------TTC

SSR120 CCCCATCACCACCTCCTGATATTCTTGTTTTGCAGCAGCAGCAGCAGCAGCAGCAGCTTC

********************************************* ***

SSR108 CTCTTTGTTCTTGTTACAGGCTCTCTGAAGGTTGCTTAAGGACTCAGATATGCCTCTCCC

SSR111 CTCTTTGTTCTTGTTACAGGCTCTCTGAAGGTTGCTTAAGGACTCAGATATGCCTCTCCC

SSR114 CTCTTTGTTCTTGTTACAGGCTCTCTGAAGGTTGCTTAAGGACTCAGATATGCCTCTCCC

SSR120 CTCTTTGTTCTTGTTACAGGCTCTCTGAAGGTTGCTTAAGGACTCAGATATGCCTCTCCC

************************************************************

SSR108 TTTGGCATTTCCTGATGATGATGAAGATCCAATCTTTAACTTTAAGCACTCAGGTATGCC

SSR111 TTTGGCATTTCCTGATGATGATGAAGATCCAATCTTTAACTTTAAGCACTCAGGTATGCC

SSR114 TTTGGCATTTCCTGATGATGATGAAGATCCAATCTTTAACTTTAAGCACTCAGGTATGCC

SSR120 TTTGGCATTTCCTGATGATGATGAAGATCCAATCTTTAACTTTAAGCACTCAGGTATGCC

************************************************************

SSR108 ATTGAAGGCATAGAGAGAAGCAGAGAACTTTCTTTCATATTTCATTCTCTTCATGGCTGC

SSR111 ATTGAAGGCATAGAGAGAAGCAGAGAACTTTCTTTCATATTTCATTCTCTTCATGGCTGC

SSR114 ATTGAAGGCATAGAGAGAAGCAGAGAATTTTCTTTCATATTTCATTCTCTTCATGGCTGC

SSR120 ATTGAAGGCATAGAGAGAAGCAGAGAACTTTCTTTCATATTTCATTCTCTTCATGGCTGC

*************************** ********************************

SSR108 TCTTAGCATTGAAGGCTCATCTGCATAGGTCTCTGGCTCCACTGGCTCCTCTATCTCCTC

SSR111 TCTTAGCATTGAAGGCTCATCTGCATAGGTCTCTGGCTCCACTGGCTCCTCTATCTCCTC

SSR114 TCTTAGCATTGAAGGCTCATCTGCATAGGTCTCTGGCTCCACTGGCTCCTCTATCTCCTC

SSR120 TCTTAGCATTGAAGGCTCATCTGCATAGGTCTCTGGCTCCACTGGCTCCTCTATCTCCTC

************************************************************

SSR108 AGCAATCTCAAAAGGAGATGGAGAGTAGTAGTTGGATGCTCCAATATCATCCACAAACTG

SSR111 AGCAATCTCAAAAGGAGATGGAGAGTAGTAGTTGGATGCTCCAATATCATCCACAAACTG

SSR114 AGCAATCTCAAAAGGAGATGGAGAGTAGTAGTTGGATGCTCCAATATCATCCACAAACTG

SSR120 AGCAATCTCAAAAGGAGATGGAGAGTAGTAGTTGGATGCTCCAATATCATCCACAAACTG

************************************************************

SSR108 GAAATCAATCACACCAACTTGATCATTGACTTCTTCTTCTTCTTGATCCATATCTTCCTC

SSR111 GAAATCAATCACACCAACTTGATCATTGACTTCTTCTTCTTCTTGATCCATATCTTCCTC

SSR114 GAAATCAATCACACCAACTTGATCATTGACTTCTTCTTCTTCTTGATCCATATCTTCCTC

SSR120 GAAATCAATCACACCAACTTGATCATTGACTTCTTCTTCTTCTTGATCCATATCTTCCTC

************************************************************

SSR108 TGATCCTTCACCGACAAAATTTGCATCCCCGCCACTGTAAGTCTGTGCCACTTCACTCAA

SSR111 TGATCCTTCACCGACAAAATTTGCATCCCCGCCACTGTAAGTCTGTGCCACTTCACTCAA

SSR114 TGATCCTTCACCGACAAAATTTGCATCCCCGCCACTGTAAGTCTGTGCCACTTCACTCAA

SSR120 TGATCCTTCACCGACAAAATTTGCATCCCCGCCACTGTAAGTCTGTGCCACTTCACTCAA

************************************************************

SSR108 ATTGCCATAGTCCATTTGATCAGTTTCTCTTGCAGTAGAGTACTCTTCCTTCTCAGTCAA

SSR111 ATTGCCATAGTCCATTTGATCAGTTTCTCTTGCAGTAGAGTACTCTTCCTTCTCAGTCAA

SSR114 ATTTCCATAGTCCATTTGATCAGTTTCTCTTGCAGTAGAGTACTCTTCCTTCTCAGTCAA

SSR120 ATTGCCATAGTCCATTTGATCAGTTTCTCTTGCAGTAGAGTACTCTTCCTTCTCAGTCAA

*** ********************************************************

SSR108 GCAAGATTCAAAGCTTGATGTCATGTAGTCAATGCTACTTGCACTAATGATGTCATGATT

SSR111 GCAAGATTCAAAGCTTGATGTCATGTGGTCAATGCTACTTGCACTAATGATGTCATGATT

SSR114 GCAAGATTCAAAGCTTGATGTCATGTAGTCAATGCTACTTGCACTAATGATGTCATGATT

SSR120 GCAAGATTCAAAGCTTGATGTCATGTAGTCAATGCTACTTGCACTAATGATGTCATGATT

************************** *********************************

SSR108 GTTGCTTGTGATGTAATCTTCAGGATCATTAAGGAAATCAGTGTATTGGGTTTCTGAGAA

SSR111 GTTGCTTGTGATGTAATCTTCAGGATCATTAAGGAAATCAGTGTATTGGGTTTCTGAGAA

SSR114 GTTGCTTGTGATGTAATCTTCAGGATCATTAAGGAAATCAGTGTATTGGGTTTCTGAGAA

SSR120 GTTGCTTGTGATGTAATCTTCAGGATCATTAAGGAAATCAGTGTATTGGGTTTCTGAGAA

************************************************************

SSR108 ATCTGCTCCTGCTTCTTCTTCCTGATGATCAGCATGTTGCTGCATTTGATGGTAGTTGAT

SSR111 ATCTGCTCCTGCTTCTTCTTCCTGATGATCAGCATGTTGCTGCATTTGATGGTGGTTGAT

SSR114 ATCTGCTCCTGCTTCTTCTTCCTGATGATCAGCATGTTGCTGCATTTGATGGTGGTTGAT

SSR120 ATCTGCTCCTGCTTCTTCTTCCTGATGATCAGCATGTTGCTGCATTTGATGGTGGTTGAT

***************************************************** ******

SSR108 AGGCAAAGGAGCTGAAGGAGCAGAACAAGAGCTGCTGTTGTTTCTTGCTTTGAGCCTCTG

SSR111 AGGCAAAGGAGCTGAAGGAGCAGAACAAGAGCTGCTGTTGTTTCTTGCTTTGAGCCTCTG

SSR114 AGGCAAAGGAGCTGAAGGAGCAGAACAAGAGCTGCTGTTGTTTCTTGCTTTGAGCCTCTG

SSR120 AGGCAAAGGAGCTGAAGGAGCAGAACAAGAGCTGCTGTTGTTTCTTGCTTTGAGCCTCTG

************************************************************

SSR108 CAGGAGAAGGTTTGTGATCTTTGAGGGAAGTGCTGGTGTTGAAGATGGTGAATGTGAGCA

SSR111 CAGGAGAAGGTTTGTGATCTTTGAGGGAAGTGCTGGTGTTGAAGATGGTGAATGTGAGCA

SSR114 CAGGAGAAGGTTTGTGATCTTTGAGGGAAGTGCTGGTGTTGAAGATGGTGAATGTGAGCA

SSR120 CAGGAGAAGGTTTGTGATCTTTGAGGGAAGTGCTGGTGTTGAAGATGGTGAATGTGAGCA

************************************************************

SSR108 AGGCCAGAAATTGGTTCGAGTATTGGCACCGCGGAGCAAGCAAGCAGCCTCATCATAGGC

SSR111 AGGCCAGAAATTGGTTCGAGTATTGGCACCGCGGAGCAAGCAAGCAGCCTCATCATAGGC

SSR114 AGGCCAGAAATTGGTTCGAGTATTGGCACCGCGGAGCAAGCAAGCAGCCTCATCATAGGC

SSR120 AGGCCAGAAATTGGTTCGAGTATTGGCACCGCGGAGCAAGCAAGCAGCCTCATCATAGGC

************************************************************

SSR108 CCTGGCTGCTTCCTCAGCAGTGTCAAATGTGCCCAACCACACTCTTATCTTTTGAATGGT

SSR111 CCTGGCTGCTTCCTCAGCAGTGTCAAATGTGCCCAACCACACTCTTATCTTTTGAATGGT

SSR114 CCTGGCTGCTTCCTCAGCAGTGTCAAATGTGCCCAACCACACTCTTATCTTTTGAATGGT

SSR120 CCTGGCTGCTTCCTCAGCAGTGTCAAATGTGCCCAACCACACTCTTATCTTTTGAATGGT

************************************************************

SSR108 GTCCTTAATCTCAGCCACCCATCTTCCGGAGGGTCTTTGGCGGACACCGACAAAGCGTTT

SSR111 GTCCTTAATCTCAGCCACCCATCTTCCGGAGGGTCTTTGGCGGACACCGACAAAGCGTTT

SSR114 GTCCTTAATCTCAGCCACCCATCTTCCGGAGGGTCTTTGGCGGACACCGACAAAGCGTTT

SSR120 GTCCTTAATCTCAGCCACCCATCTTCCGGAGGGTCTTTGGCGGACACCGACAAAGCGTTT

************************************************************

SSR108 TCGAGCTCTTCGTGCTCCTCCTAGCTCTGCAGCAGCCGAAGCTTCCTTCACCATCTCATC

SSR111 TCGAGCTCTTCGTGCTCCTCCTAGCTCTGCAGCAGCCGAAGCTTCCTTCACCATCTCATC

SSR114 TCGAGCTCTTCGTGCTCCTCCTAGCTCTGCAGCAGCCGAAGCTTCCTTCACCATCTCATC

SSR120 TCGAGCTCTTCGTGCTCCTCCTAGCTCTGCAGCAGCCGAAGCTTCCTTCACCATCTCATC

************************************************************

SSR108 CCAAGCCATGGTTCCCTCACTTGAACTCCTGTCTTCCACTCCATCACTAACTTTTCTCTT

SSR111 CCAAGCCATGGTTCCCTCACTTGAACTCCTGTCTTCCACTCCATCACTAACTTTTCTCTT

SSR114 CCAAGCCATGGTTCCCTCACTTGAACTCCTGTCTTCCACTCCATCACTAACTTTTCTCTT

SSR120 CCAAGCCATGGTTCCCTCACTTGAACTCCTGTCTTCCACTCCATCACTAACTTTTCTCTT

************************************************************

SSR108 CCTTGCCAT

SSR111 CCTTGCCAT

SSR114 CCTTGCCAT

SSR120 CCTTGCCAT

*********
