# Supplementary material for: Genetic dissection of apricot fruit skin color (Prunus armeniaca L.) using SNP and SSR molecular markers
Source: Mol Breed. 2026 May 22;46(6):50. doi: 10.1007/s11032-026-01674-5 (PMC13197536; doi:10.1007/s11032-026-01674-5)
Supplement: Supplementary file 5 — Supplementary file5 (DOCX 120 KB) [file 11032_2026_1674_MOESM5_ESM.docx]

**Table S5.**

**Protein sequences**

1. **Protein sequence with SSR size of 108 pb (3’5’ frame 1)**

MARKRKVSDGVEDRSSSEGTMAWDEMVKEASAAAELGGARRARKRFVGVRQRPSGRWVAEIKDTIQKIRVWLGTFDTAEEAARAYDEAACLLRGANTRTNFWPCSHSPSSTPALPSKITNLLLQRLKARNNSSSCSAPSAPLPINYHQMQQHADHQEEEAGADFSETQYTDFLNDPEDYITSNNHDIISASSIDYMTSSFESCLTEKEEYSTARETDQMDYGNLSEVAQTYSGGDANFVGEGSEEDMDQEEEEVNDQVGVIDFQFVDDIGASNYYSPSPFEIAEEIEEPVEPETYADEPSMLRAAMKRMKYERKFSASLYAFNGIPECLKLKIGSSSSSGNAKGRGISESLSNLQRACNKNKEEAAAAAKQEYQEVVMGKKEEEETQLSSMDISLSSDGELSLWSSLDLQPICFLSTN-

1. **Protein sequence with SSR size of 111 pb (3’5’ frame 1)**

MARKRKVSDGVEDRSSSEGTMAWDEMVKEASAAAELGGARRARKRFVGVRQRPSGRWVAEIKDTIQKIRVWLGTFDTAEEAARAYDEAACLLRGANTRTNFWPCSHSPSSTPALPSKITNLLLQRLKARNNSSSCSAPSAPLPINHHQMQQHADHQEEEAGADFSETQYTDFLNDPEDYITSNNHDIISASSIDHMTSSFESCLTEKEEYSTARETDQMDYGNLSEVAQTYSGGDANFVGEGSEEDMDQEEEEVNDQVGVIDFQFVDDIGASNYYSPSPFEIAEEIEEPVEPETYADEPSMLRAAMKRMKYERKFSASLYAFNGIPECLKLKIGSSSSSGNAKGRGISESLSNLQRACNKNKEEAAAAAAKQEYQEVVMGKKEEEETQLSSMDISLSSDGELSLWSSLDLQPICFLSTN-

1. **Protein sequence with SSR size of 114 pb (3’5’ frame 1)**

MARKRKVSDGVEDRSSSEGTMAWDEMVKEASAAAELGGARRARKRFVGVRQRPSGRWVAEIKDTIQKIRVWLGTFDTAEEAARAYDEAACLLRGANTRTNFWPCSHSPSSTPALPSKITNLLLQRLKARNNSSSCSAPSAPLPINHHQMQQHADHQEEEAGADFSETQYTDFLNDPEDYITSNNHDIISASSIDYMTSSFESCLTEKEEYSTARETDQMDYGNLSEVAQTYSGGDANFVGEGSEEDMDQEEEEVNDQVGVIDFQFVDDIGASNYYSPSPFEIAEEIEEPVEPETYADEPSMLRAAMKRMKYERKFSASLYAFNGIPECLKLKIGSSSSSGNAKGRGISESLSNLQRACNKNKEEAAAAAAAKQEYQEVVMGKKEEEETQLSSMDISLSSDGELSLWSSLDLQPICFLSTN-

1. **Protein sequence with SSR size of 120 pb (3’5’ frame 1)**

MARKRKVSDGVEDRSSSEGTMAWDEMVKEASAAAELGGARRARKRFVGVRQRPSGRWVAEIKDTIQKIRVWLGTFDTAEEAARAYDEAACLLRGANTRTNFWPCSHSPSSTPALPSKITNLLLQRLKARNNSSSCSAPSAPLPINHHQMQQHADHQEEEAGADFSETQYTDFLNDPEDYITSNNHDIISASSIDYMTSSFESCLTEKEEYSTARETDQMDYGNLSEVAQTYSGGDANFVGEGSEEDMDQEEEEVNDQVGVIDFQFVDDIGASNYYSPSPFEIAEEIEEPVEPETYADEPSMLRAAMKRMKYERKFSASLYAFNGIPECLKLKIGSSSSSGNAKGRGISESLSNLQRACNKNKEEAAAAAAAAAKQEYQEVVMGKKEEEETQLSSMDISLSSDGELSLWSSLDLQPICFLSTN-

**CLUSTAL 2.1 multiple sequence alignment**

SSR108 MARKRKVSDGVEDRSSSEGTMAWDEMVKEASAAAELGGARRARKRFVGVRQRPSGRWVAE

SSR111 MARKRKVSDGVEDRSSSEGTMAWDEMVKEASAAAELGGARRARKRFVGVRQRPSGRWVAE

SSR114 MARKRKVSDGVEDRSSSEGTMAWDEMVKEASAAAELGGARRARKRFVGVRQRPSGRWVAE

SSR120 MARKRKVSDGVEDRSSSEGTMAWDEMVKEASAAAELGGARRARKRFVGVRQRPSGRWVAE

************************************************************

SSR108 IKDTIQKIRVWLGTFDTAEEAARAYDEAACLLRGANTRTNFWPCSHSPSSTPALPSKITN

SSR111 IKDTIQKIRVWLGTFDTAEEAARAYDEAACLLRGANTRTNFWPCSHSPSSTPALPSKITN

SSR114 IKDTIQKIRVWLGTFDTAEEAARAYDEAACLLRGANTRTNFWPCSHSPSSTPALPSKITN

SSR120 IKDTIQKIRVWLGTFDTAEEAARAYDEAACLLRGANTRTNFWPCSHSPSSTPALPSKITN

************************************************************

SSR108 LLLQRLKARNNSSSCSAPSAPLPINYHQMQQHADHQEEEAGADFSETQYTDFLNDPEDYI

SSR111 LLLQRLKARNNSSSCSAPSAPLPINHHQMQQHADHQEEEAGADFSETQYTDFLNDPEDYI

SSR114 LLLQRLKARNNSSSCSAPSAPLPINHHQMQQHADHQEEEAGADFSETQYTDFLNDPEDYI

SSR120 LLLQRLKARNNSSSCSAPSAPLPINHHQMQQHADHQEEEAGADFSETQYTDFLNDPEDYI

*************************:**********************************

SSR108 TSNNHDIISASSIDYMTSSFESCLTEKEEYSTARETDQMDYGNLSEVAQTYSGGDANFVG

SSR111 TSNNHDIISASSIDHMTSSFESCLTEKEEYSTARETDQMDYGNLSEVAQTYSGGDANFVG

SSR114 TSNNHDIISASSIDYMTSSFESCLTEKEEYSTARETDQMDYGNLSEVAQTYSGGDANFVG

SSR120 TSNNHDIISASSIDYMTSSFESCLTEKEEYSTARETDQMDYGNLSEVAQTYSGGDANFVG

**************:*********************************************

SSR108 EGSEEDMDQEEEEVNDQVGVIDFQFVDDIGASNYYSPSPFEIAEEIEEPVEPETYADEPS

SSR111 EGSEEDMDQEEEEVNDQVGVIDFQFVDDIGASNYYSPSPFEIAEEIEEPVEPETYADEPS

SSR114 EGSEEDMDQEEEEVNDQVGVIDFQFVDDIGASNYYSPSPFEIAEEIEEPVEPETYADEPS

SSR120 EGSEEDMDQEEEEVNDQVGVIDFQFVDDIGASNYYSPSPFEIAEEIEEPVEPETYADEPS

************************************************************

SSR108 MLRAAMKRMKYERKFSASLYAFNGIPECLKLKIGSSSSSGNAKGRGISESLSNLQRACNK

SSR111 MLRAAMKRMKYERKFSASLYAFNGIPECLKLKIGSSSSSGNAKGRGISESLSNLQRACNK

SSR114 MLRAAMKRMKYERKFSASLYAFNGIPECLKLKIGSSSSSGNAKGRGISESLSNLQRACNK

SSR120 MLRAAMKRMKYERKFSASLYAFNGIPECLKLKIGSSSSSGNAKGRGISESLSNLQRACNK

************************************************************

SSR108 NKEE----AAAAAKQEYQEVVMGKKEEEETQLSSMDISLSSDGELSLWSSLDLQPICFLS

SSR111 NKEE---AAAAAAKQEYQEVVMGKKEEEETQLSSMDISLSSDGELSLWSSLDLQPICFLS

SSR114 NKEE--AAAAAAAKQEYQEVVMGKKEEEETQLSSMDISLSSDGELSLWSSLDLQPICFLS

SSR120 NKEEAAAAAAAAAKQEYQEVVMGKKEEEETQLSSMDISLSSDGELSLWSSLDLQPICFLS

****::::****************************************************

SSR108 TN-

SSR111 TN-

SSR114 TN-

SSR120 TN-

**
